# From Detection to Maps: A Review of Automated Urban Tree Mapping Using UAV and High-Resolution Satellite Data

**DOI:** 10.3390/jimaging12080358

**Published:** 2026-08-06

**Authors:** Syndar Satbayev, Didar Yedilkhan, Aruzhan Shoman, Azamat Serek, Mohammad Shadab Khan

**Affiliations:** 1 School of Artificial Intelligence and Data Science, Astana IT University, Astana 010000, Kazakhstan; satbayev.syndar@astanait.edu.kz (S.S.); azamat.serek@astanait.edu.kz (A.S.); 2 Smart City Research Center, Astana IT University, Astana 010000, Kazakhstan; 3 AgroTech Research and Innovation Center, Astana IT University, Astana 010000, Kazakhstan; 4 Faculty of Computing and Informatics (FCI), Multimedia University, Cyberjaya 63100, Malaysia; shadab.khan@mmu.edu.my

**Keywords:** urban tree, deep learning, object detection, semantic segmentation, UAV, satellite, mapping, tree health assessment

## Abstract

Urban tree mapping is necessary in environmental sustainability and climate change mitigation, and it depends heavily on the individual tree recognition and canopy segmentation to analyze city green cover. This systematic review discusses recent developments in the 2014–2026 mapping of these trees with the use of UAVs and high-resolution satellite imagery. Our preliminary selection of 4148 records reduced to 101 eligible publications following a systematic screening and synthesis of the records, assessed the efficiency of deep learning models such as Convolutional Neural Networks and Vision Transformers in processing various source images. We also compare object detection and semantic segmentation to see which one is more competent to deal with typical urban challenges, including overlapped canopies and building shadows. According to the reviewed studies, UAV-based models generally achieve higher spatial accuracy than satellite-based approaches for individual tree detection and crown delineation, with reported average Intersection over Union (IoU) values of approximately 70–75%, whereas satellite imagery provides superior spatial coverage for large-scale urban forest monitoring. Lastly, we present a research roadmap to address the existing weaknesses such as geographic bias, which propels the research direction towards multimodal data fusion and Foundation Models to sustain consistent, large-scale urban forest monitoring.

## 1. Introduction

### 1.1. Importance of Urban Trees

Urban trees are fundamental to the environmental health of our cities, helping to soften the blow of climate change and making urban spaces significantly more livable. Beyond simply absorbing carbon dioxide, they actively cool the air through shade and evaporation, directly combating the Urban Heat Island effect while filtering out harmful pollutants [1,2]. In fact, adding greenery to densely built neighborhoods can drop surface temperatures by a noticeable 1–3 °C [2]. Trees also quietly support city infrastructure; their root systems and canopy cover reduce the strain on stormwater drains, enhance soil permeability, and even prolong the life of paved roads by keeping them shaded [3]. The financial impact of these ecosystem services is massive; Nowak et al. estimated that climate regulation, cleaner air, and better public health generate billions of dollars in annual economic benefits [4]. Because our cities are growing rapidly alongside increasingly unpredictable climate patterns, maintaining accurate, scalable, and up-to-date records of our urban forests has never been more critical [2].

### 1.2. Challenges in Automated Tree Mapping

Automated tree mapping is not without technical challenges that are unique to the process, though it has its obvious advantages. Although drones take remarkably detailed photos, they can fly over a small area of land at the time and are completely vulnerable to the weather conditions [5,6,7]. Satellites scan large areas with ease, but their images are frequently too coarse to be depended upon to identify individual trees. Also, thick shadows of the buildings and a small amount of spectral information can significantly bias the analysis [5,8].

The detection software is also ineffective. In the case of trees, which are closely spaced, such as in a tightly packed city, it is very hard, because of the overlapping canopies, to determine where one tree starts and another one begins [9]. Also, the variations in the species and the spectral similarity of the vegetation to the rest of the green objects result in misclassifications and false positive identifications [9,10]. Due to such strictly reactive sensitivities, AI systems tend to fall over when the environment changes. As it has also been noted in numerous studies, the accuracy of a model often decreases as soon as it is presented with a new season, altered weather patterns, or just a different geographic region [11,12]. Before the emergence of deep learning, many of these challenges were addressed using conventional remote sensing, photogrammetric, and image-analysis techniques, which laid the methodological foundation for modern AI-based approaches.

### 1.3. Evolution of Automated Tree Mapping Before Deep Learning

Before the widespread adoption of deep learning, automated tree mapping was primarily based on conventional remote sensing, photogrammetric, and image-analysis techniques [13,14,15,16]. Early tree mapping and forest inventory primarily relied on field-based measurements and conventional geomatics techniques. Traditional forest inventories focused on measuring diameter at breast height (DBH), tree height, and subsequently estimating stem volume, above-ground biomass, and other forest inventory attributes. With the development of remote sensing, these methods were progressively complemented by aerial photogrammetry and UAV-based Structure-from-Motion (SfM), as well as laser scanning technologies including terrestrial laser scanning (TLS), mobile laser scanning (MLS), Airborne Laser Scanning (ALS), and more recently UAV laser scanning (ULS). These technologies enabled accurate three-dimensional reconstruction of forest structure and improved the estimation of key inventory attributes, including crown dimensions, stem volume, and above-ground biomass, providing the operational foundation for modern forest inventory and supporting subsequent advances in automated tree mapping [17,18,19]. Common approaches included object-based image analysis (OBIA), local maxima filtering, template matching, watershed segmentation, region growing, and Canopy Height Models (CHMs) derived from LiDAR data [13,14,15]. Spectral vegetation indices, such as NDVI, were frequently used to support vegetation discrimination and tree extraction [13]. These approaches formed the methodological foundation of operational forest inventory and early urban tree mapping for many years [14,16]. Although effective under controlled conditions, their performance often depended on manually designed features and carefully tuned parameters, making them sensitive to complex urban backgrounds, overlapping crowns, illumination variability, and differences between study areas [14]. The emergence of Convolutional Neural Networks after 2014 significantly improved automatic feature learning and detection performance, overcoming many of the limitations associated with handcrafted feature extraction while improving model generalization and accuracy [20].

### 1.4. AI-Based Methods

Recently, deep learning techniques, especially Convolutional Neural Networks (CNNs), have become the tools of choice when it comes to mapping urban trees in an automated fashion. Semantic segmentation Architectures such as U-Net and DeepLabv3+ are capable of extracting high-quality tree canopy data directly out of aerial and satellite data. These neural networks demonstrate easy superiority over conventional algorithms such as object-based image analysis when it comes to the detection of tree crowns [21]. To contextualize this, a study reported U-Net reaching a staggering Intersection over Union (IoU) of approximately 0.96 which was significantly higher than the classical method at 0.49 [21]. To further cement this change in direction, a recent experiment that compared five various CNNs to one another returned an F1-score of about 91% on the task of segmenting urban vegetation, demonstrating just how accurate deep learning methods have become at extracting accurate information on urban vegetation [22].

In addition to mapping canopy cover in general, scientists are already applying CNN-based object-detection algorithms to locate specific trees, which are camouflaged in complicated urban environments. YOLO-based single-stage detectors have proven particularly effective at localizing trees in high-resolution aerial and satellite imagery, with most models achieving accuracy scores above 90% [12,23]. Another notable example is a lightweight YOLOv4-Lite model with a MobileNet backbone, which resulted in 96–98% detection rates in a variety of environments, such as street curbs, gardens, and dense plantation [20]. Two-stage detectors are also taking their toll. An adapted Faster R-CNN with a Swin Transformer backbone was significantly better than its classical ResNet equivalent, with an AP50 of approximately 0.70 when searching street trees in orthophotos [24]. Going even further, past research achieved the highest possible performance of 86–92% and 77–83% Accuracy and Recall respectively by either using heavier models such as YOLOv5x or combining the results of several neural networks intelligently [25].

Despite these developments, the intricate city background is a formidable challenge. The tightness of the buildings, the overlay of shadows, the messy trees, and the misleading areas of other similar vegetation continue to stump automated systems. An example is the heavy shadows cast by skyscrapers that can harshly undermine the precision of the tree segmentation on the street, despite the recent deep learning models at work [8]. The majority of errors can be attributed to a total absence of small or partially obscured trees, or fooling because of the false positives of a dark shadow or a shrub or a patch of grass and an adult tree because they are of similar spectral signature [22,25]. To make such unpredictable conditions hard on these algorithms, the current network architectures are becoming more and more baked with attention mechanisms and multi-scale contextual modules to have a better comprehension of the surrounding environment [8,24].

As an example, a dual multi-scale network processing both standard RGB and NDVI data as well as a channel attention mechanism were tested by researchers. Although this arrangement outcompetes the traditional CNN models in terms of segmentation accuracy, it remains having issues with overlapping branches and intense shadows [8]. To overcome these bottlenecks, teams are becoming more and more an amalgamation of completely dissimilar types of information. By integrating top-down aerial or satellite images with ground-level images, such as Google Street View panoramas, which have become an important source of urban GIS information [26], into a single deep learning model, one can locate trees on the street that cannot be seen at all by the buildings at the top. This piece of creative workaround was effective to identify approximately 79% of the trees in the street, and their locations with an average error of only 0.6 m [25].

With a more nutritious diet of multispectral data (including near-infrared bands and NDVI) or a LiDAR added to the neural network also hones the ability to distinguish between a tree canopy and other urban elements, particularly in shadowy or high-density regions [8,24]. To eradicate the remaining blind spots, certain research proposes to introduce the drones (UAVs) into the picture, sweeping over the enclosed courtyards and other narrow areas that are not covered by satellites and street cameras [25]. The discipline is also shifting away from not just plotting trees on a map, but on to the analysis of trees themselves. More recent installations are becoming based on instance segmentation models such as Mask R-CNN to follow the precise edges of treetops and approximate their physical dimensions, even in densely populated neighborhoods [11,22]. In conclusion, it is evident that AI is capable of mapping urban trees with unprecedented accuracy and scale, which is possible through these deep learning breakthroughs. The next challenge, however, will be to refine these systems in order to remove chaotic backgrounds of cities and to successfully incorporate various data streams to create a single entire picture of the urban forest.

### 1.5. Research Motivation and Contributions

Even with the major leaps forward we have seen lately, today’s automated systems for mapping urban trees still hit a wall when it comes to scaling up, working across different cities, or seamlessly blending various types of imagery. Models that perform perfectly in one location often fall apart when tested in a new geographic setting. On top of that, researchers struggle to bridge the gap between drone and satellite data, a missing link that currently prevents us from capturing both fine spatial detail and massive area coverage simultaneously. To make matters worse, the opaque, “black-box” nature of deep learning, combined with a frustrating lack of public, annotated datasets, makes these systems difficult to reproduce and adopt in the real world.

To address these issues, this review systematically breaks down the progress made in automated urban tree mapping between 2014 and 2026, focusing specifically on drone and high-resolution satellite imagery. By examining 101 different publications, we offer a clear, structured look at the latest deep learning techniques, datasets, and practical workflows. We pay special attention to how Convolutional Neural Networks like U-Net and YOLO have evolved, alongside new strategies for merging data across different scales. Drawing from this evidence, we pinpoint the most critical gaps in current research and lay out a clear roadmap for building resilient, large-scale urban tree mapping systems in the future.

## 2. Materials and Methods

To maintain a rigorous and transparent methodology, we structured this review according to established systematic review guidelines. The entire process unfolded in four distinct stages: conducting a thorough literature search, carefully screening the results for eligibility, extracting the core data, and finally bringing it all together through a qualitative synthesis of the chosen studies.

### 2.1. Search Strategy

We systematically scanned major digital libraries and bibliographic databases to track down relevant research on mapping, detecting, segmenting and monitoring trees via drones and high-resolution satellites. Wrapping up on 12 February 2026, our search strategy linked five core concepts:

1. The target object (trees and tree crowns);
2. The task (detection, segmentation, counting, and mapping/georeferencing);
3. The data source (UAV/drone imagery and high-resolution satellite imagery);
4. AI approaches (deep learning and common model families);
5. The extended monitoring focus, where relevant, including tree health assessment and temporal canopy change.

To cast a wide net, our search queries incorporated a broad mix of keywords and synonyms. We targeted the subjects with terms like “individual tree,” “tree crown,” “canopy,” “urban tree,” and “street tree,” while defining the specific tasks using words such as “detection,” “object detection,” “segmentation” (including both semantic and instance), “tree counting,” “mapping,” and “georeferencing.” We also zeroed in on technology by including platform identifiers like “UAV,” “drone,” “unmanned aerial,” “aerial imagery,” “satellite,” and “high-resolution,” alongside core AI methods like “deep learning,” “CNN,” “YOLO,” “U-Net,” “Mask R-CNN,” and “transformer.” To capture studies relevant to tree health assessment, we also included supplementary terms such as “tree health,” “tree condition,” “canopy stress,” “change detection,” and “temporal dynamics” where these were combined with UAV/drone or high-resolution satellite imagery and AI/ML-based analysis.

Naturally, we tailored the exact Boolean operators, word truncations, and search fields (focusing on titles, abstracts, and keywords) to fit the unique quirks of each database. Finally, we did not rely solely on keyword algorithms to find our literature. To ensure no crucial research slipped past our initial queries, we manually combed through the reference lists of the papers we had already selected, a technique known as backward snowballing, to uncover any hidden, highly relevant studies.

### 2.2. Eligibility Criteria

To keep our review focused on automated tree mapping and condition-aware urban forest monitoring, we established clear eligibility rules from the very beginning. As detailed in Table 1, a study was included only if it addressed tree-related mapping, detection, segmentation, counting, georeferencing, or health assessment using UAV/drone imagery and/or high-resolution satellite imagery combined with AI/ML-based analysis.

### 2.3. Study Selection

We guided our study selection through a systematic framework, breaking the process down into several distinct stages. Initially, our database and source searches yielded a massive pool of 4148 records. By manually screening the titles and abstracts, we narrowed this down to 904 potentially relevant papers earmarked for full-text review. Before diving into the full texts, we cleaned up the dataset by filtering out 134 duplicates and dropping 76 records that simply could not be retrieved. This left us with 694 complete reports to evaluate against our strict eligibility rules (outlined in Section 2.2).

**Figure 1:**
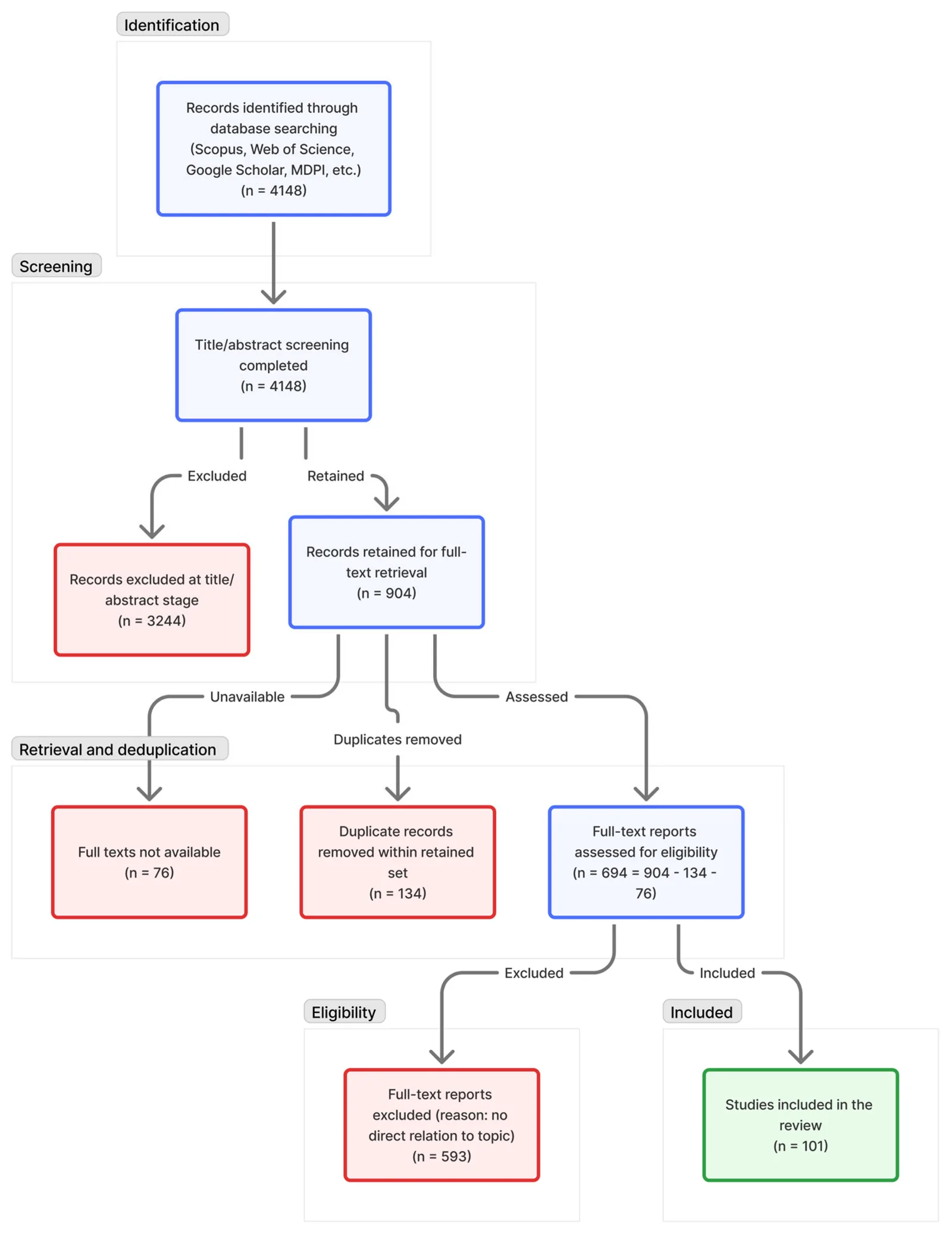
Flow diagram of the study selection process.

During this deeper evaluation, we had to discard several more papers. Some were hidden duplicates or lacked full-text access, but most were cut because they missed the mark on our core criteria—whether they lacked crucial methodological details, ignored AI/ML approaches entirely, or just did not focus on using drones and high-resolution satellites to map trees. Ultimately, exactly 101 studies survived this rigorous filtering and made it into our final synthesis, a journey we have mapped out visually in the flow diagram (Figure 1).

### 2.4. Data Extraction

To maintain consistency across all the reviewed studies, we gathered information using a standardized extraction form. For every paper, we first logged the basic bibliographic details, such as the title, publication year, journal, and DOI. We then broke down the technical methodology into several core areas. First, we identified the study’s main objective, noting whether it focused on tree detection, crown delineation, semantic and instance segmentation, counting, or large-scale mapping. Next, we recorded the data sources and geographic context, specifically looking at the use of drones or high-resolution satellites in urban and peri-urban environments. We also captured detailed dataset characteristics, including image resolution, scene counts, acquisition conditions, and the specific train, validation, and test splits. When applicable, information related to tree health assessment tasks was also extracted.

Beyond the raw data, we documented how researchers labeled their images, whether through bounding boxes, polygons, or point labels, and mapped out their entire analytical pipeline. This meant tracking everything from initial pre-processing and data augmentation to the specific deep learning architectures (like YOLO, U-Net, or Vision Transformers) and any post-processing steps. To understand how these models performed, we collected their evaluation metrics (such as Precision, Recall, F1-score, IoU, mAP, and RMSE for counting) alongside any reported baseline comparisons or ablation studies. Finally, we summarized the authors’ main findings, the limitations they faced, and the research gaps they explicitly pointed out. All these variables were systematically compiled into a structured database. Where the main text was incomplete or ambiguous, the missing information was cross-checked against other sections of the same publication before the record was finalized.

### 2.5. Quality Appraisal

To ensure our findings are completely transparent, we evaluated the reporting quality and reproducibility of each study using a custom checklist designed specifically for remote-sensing computer vision. We closely examined several key technical areas: how clearly the authors described their datasets (including sensor types, resolution, and scene details), the transparency of their labeling process, and the rigor of their data-splitting methods to prevent unintended data leakage. Beyond that, we checked whether their chosen evaluation metrics made sense, if they conducted fair baseline comparisons or sensitivity analyses, and whether they openly shared their code, models, or raw data. Ultimately, this deep dive helped us put the published results into proper context and pinpoint recurring flaws that might prevent these AI models from working reliably in the real world.

### 2.6. Data Synthesis and Analysis

The studies examined varied wildly in everything from the sensors and spatial resolutions they used to their annotation formats and evaluation metrics. Because of this massive diversity, running a strict quantitative meta-analysis simply was not feasible; instead, we opted for a comprehensive narrative synthesis. To make sense of the data, we categorized the research along three main lines: the specific task at hand (like detection, segmentation, or large-scale mapping), the type of imagery relied upon (such as drone, satellite, or a mix of both), and the underlying AI architecture (ranging from YOLO detectors and U-Net segmentations to newer transformer models). Rather than forcing a uniform evaluation standard, we assessed each model’s performance using its originally reported scores, whether that was mAP, IoU, or RMSE, and hunted for broader trends tied to specific dataset characteristics and experimental setups. Bringing it all together, we highlighted the persistent blind spots across these studies to draft a practical, forward-looking roadmap for the next generation of urban tree mapping.

## 3. Results

### 3.1. Study Characteristics

**Figure 2:**
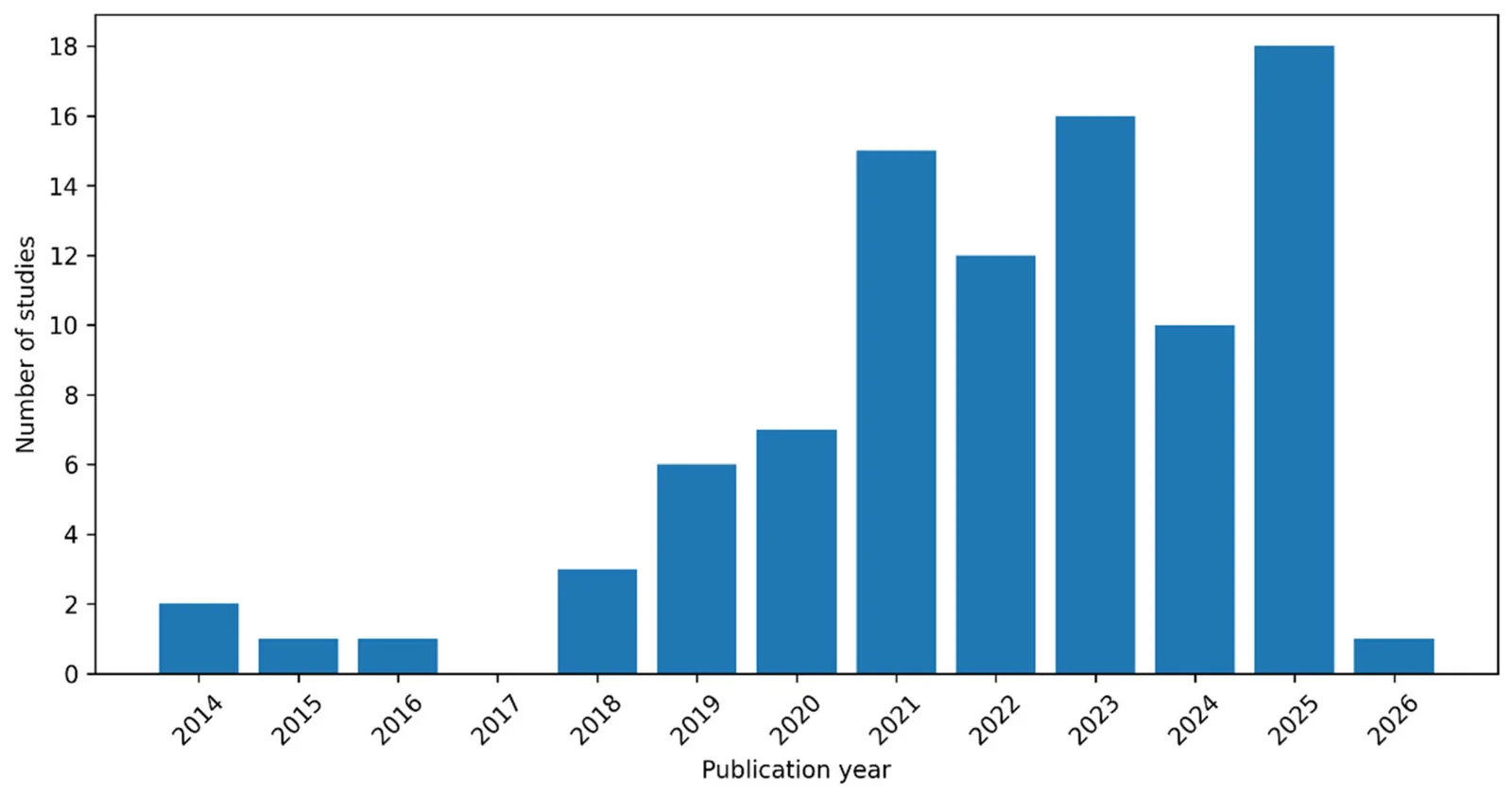
Distribution of included studies by publication year.

The research spans from 2014 to 2026, perfectly capturing the recent explosion of interest in using deep learning for tree mapping. While early pioneering work can be traced back to 2014 [27], the timeline extends right up to cutting-edge publications from 2026 [6]. However, the bulk of the literature has clearly emerged since 2020, with a noticeable peak in activity between 2021 and 2025 as the underlying technology matured [3,28]. Figure 2 provides a clear visual breakdown of this publication trend.

**Figure 3:**
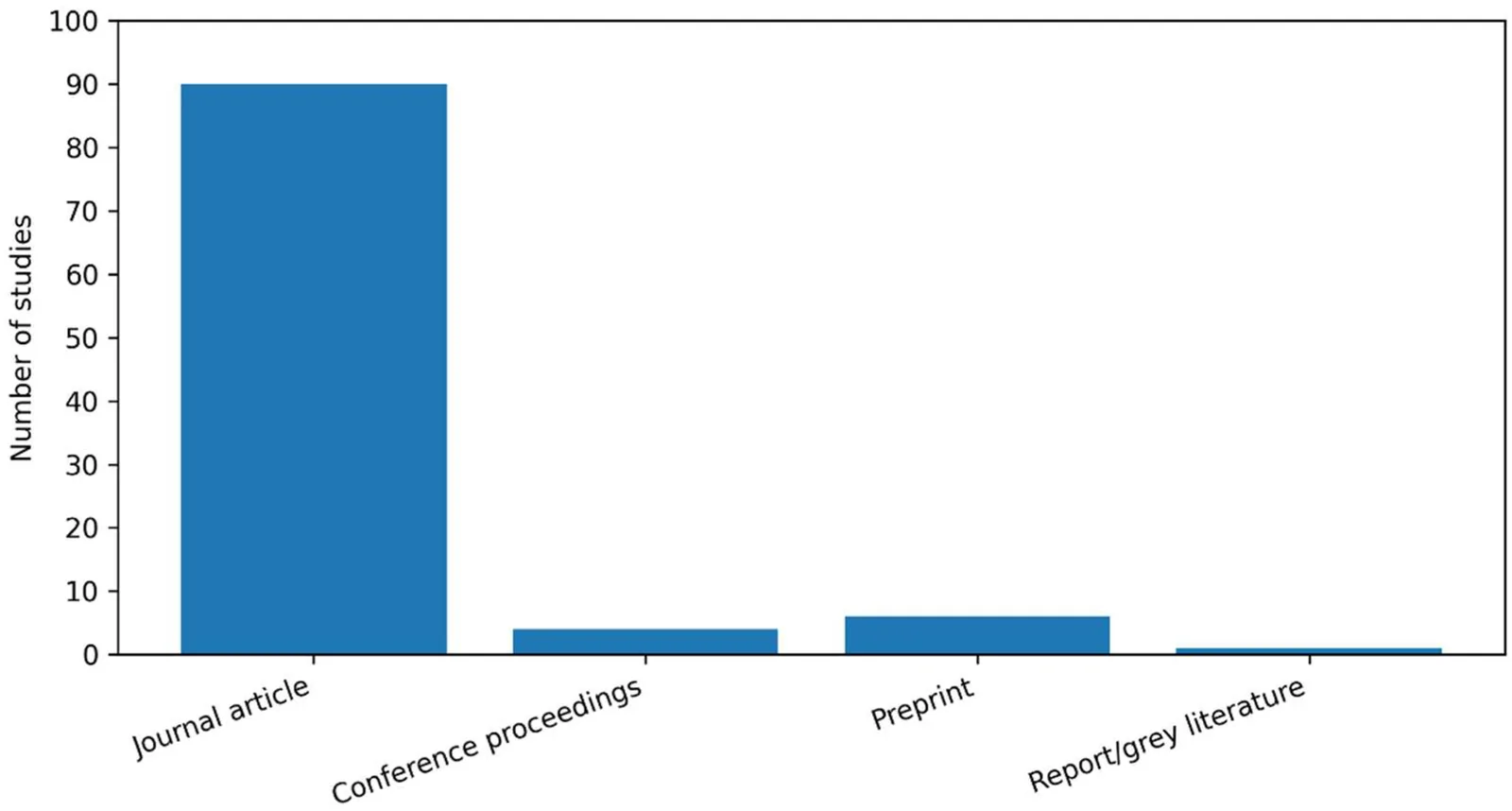
Distribution of the included studies by publication type.

When looking at publication outlets, traditional journal articles clearly dominate the landscape, especially within specialized venues like remote sensing [29,30]. Only a handful of the selected studies originate from alternative sources, such as conference proceedings [31], early preprints [1], or gray literature [32]. This heavy reliance on established journals ensures that most of our synthesized evidence has undergone rigorous peer review, with the exact distribution of these publication formats naturally reflected in Figure 3.

Although the extracted data shows inconsistent geographic reporting, several studies clearly state their locations right in the titles or methodology sections. When locations are provided, China dominates the research landscape [2,33], with Germany [6] and the United States [12] trailing behind. Researchers frequently test their models in specific major cities, such as Beijing [34], Shanghai [35], Helsinki [3], and San Francisco [12]. While this highlights a clear focus on urban environments, the sporadic geographic details underscore a pressing need for standardized reporting to truly understand how these AI models perform across different cities.

### 3.2. Tasks Taxonomy

By examining the core objectives outlined in these papers, the research naturally divides into five distinct problem categories (Table 2):

1. Detection/localization;
2. Crown segmentation and delineation (semantic vs. instance);
3. Classification (Tree Health Assessment);
4. Counting/density estimation;
5. Mapping/georeferencing (often at city or regional scale), with many papers combining two or more tasks in a single pipeline.

#### 3.2.1. Detection/Localization

**Figure 4:**
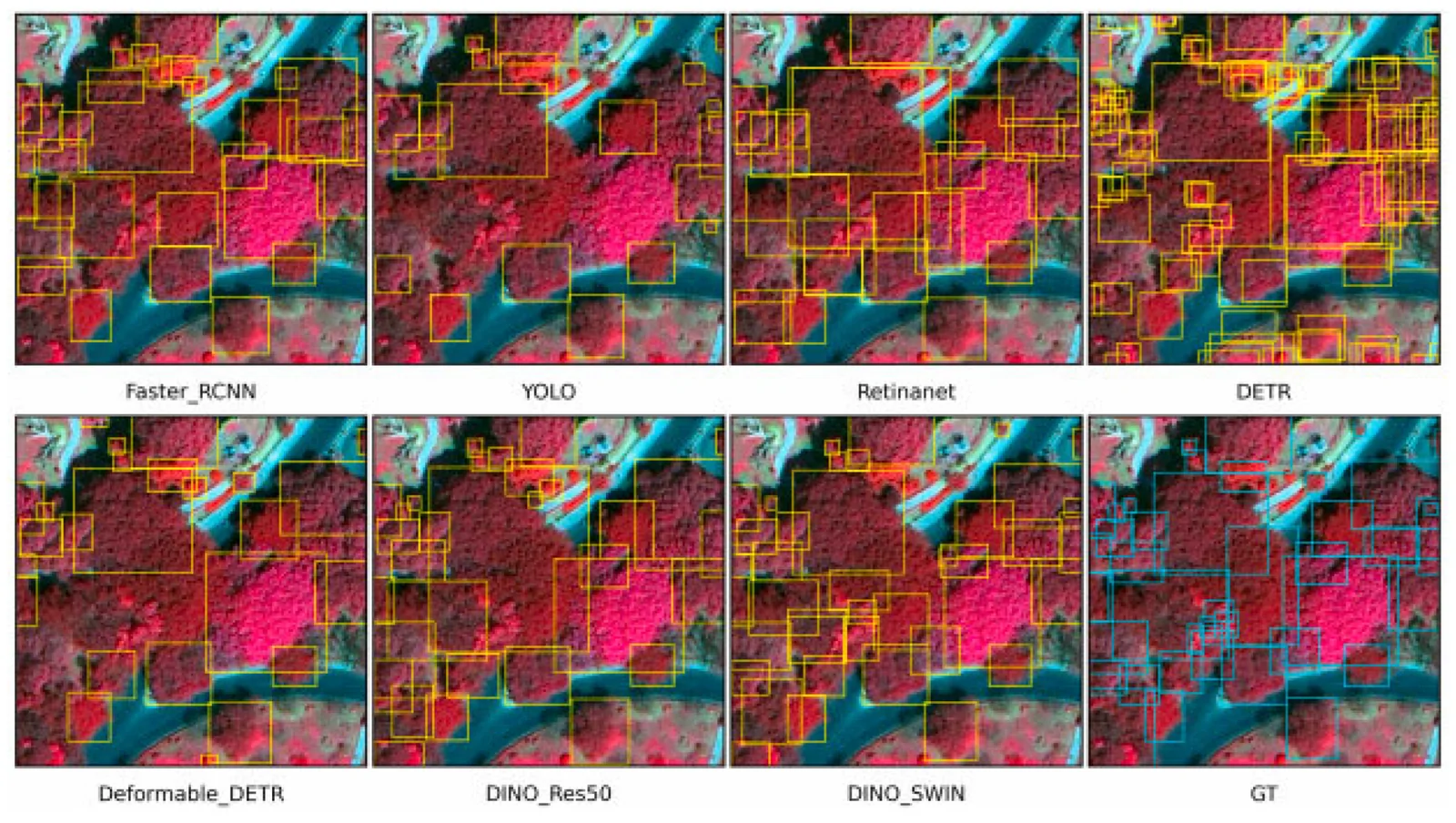
Example of the visual representation of tree detection and localization. (yellow) using five different object detection models and annotation data (cyan): trees in city parks.

Many studies focus on pinpointing individual trees or their crowns, marking them with basic bounding boxes or coordinate points. Researchers tackle this challenge using a mix of traditional remote-sensing methods, like index- or peak-based localization [32], and innovative deep learning object detectors. For large-scale mapping, fast YOLO-family architectures and newer transformer-based models like DETR have become the go-to choices. To measure how well these systems perform, evaluations typically rely on standard object-detection metrics such as Precision, Recall, F1-score, and mean Average Precision (mAP). Figure 4 illustrates a typical output of tree detection and localization, where individual tree crowns are automatically identified and spatially localized using bounding boxes generated by an object detection model [35].

Recent studies demonstrate a rapid diversification of detection frameworks. Beyond conventional YOLO- and Faster R-CNN-based approaches, recent research has explored DeepForest, Mask R-CNN, DETR, Segment Anything Model (SAM), zero-shot learning, and lightweight embedded implementations for individual tree detection under different acquisition conditions, including UAV, aerial, and satellite imagery [66,67,68,69,70,71,72,73,74,75,76,77,78]. Similar detection paradigms have also been successfully extended to other remote-sensing applications, including smoke detection [79], geo-crack detection [80], and passive radar target detection [81].

#### 3.2.2. Segmentation and Crown Delineation

**Figure 5:**
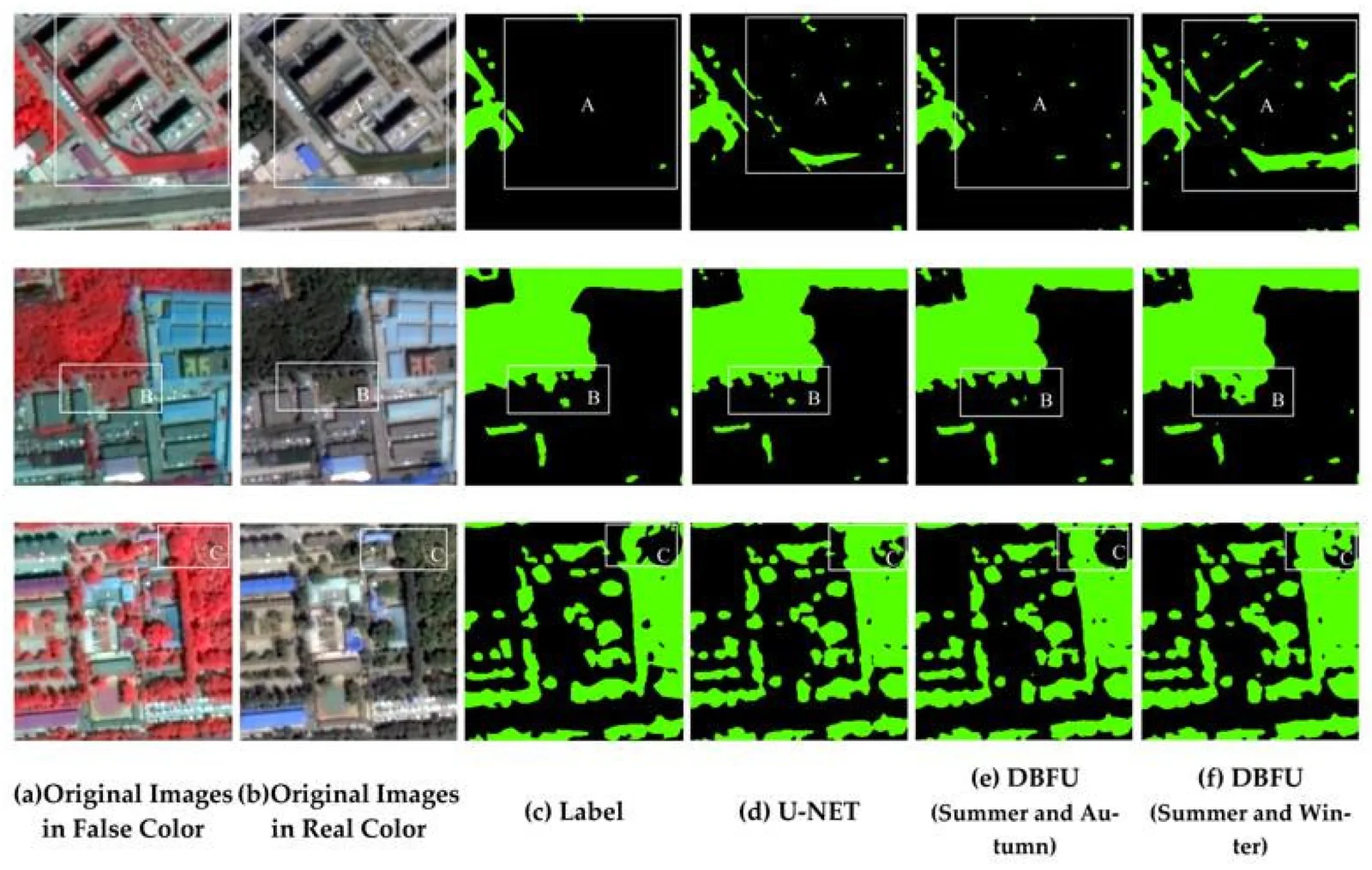
Example of the outputs of the segmentation and crown delineation. (**a**) original images in false color, (**b**) original images in real color, (**c**) ground truth, (**d**) classification results of U-NET, (**e**) classification results of DBFU, (**f**) classification results of DBFU. Rows (A–C) correspond to three different example study areas.

When the objective shifts to tracing the exact outlines of tree canopies or crowns, researchers typically turn to segmentation methods. Semantic segmentation models, often built on U-Net architectures, analyze satellite or drone images to create pixel-by-pixel masks that simply separate trees from the background [41]. On the other hand, instance segmentation takes this a step further by untangling overlapping vegetation, assigning a distinct mask to every individual tree crown [39,40,42]. To speed up this detailed work, some of the latest workflows pair initial object detection with promptable segmentation, generating these complex boundaries much more efficiently [6]. Ultimately, the success of these segmentation strategies is measured using standard metrics like IoU, the Dice/F1 score, and other related per-class evaluations. Figure 5 illustrates typical outputs of crown segmentation, highlighting the difference between semantic segmentation, which separates vegetation from the background, and instance segmentation, which delineates individual tree crowns as separate objects [41].

#### 3.2.3. Tree Health Assessment

**Figure 6:**
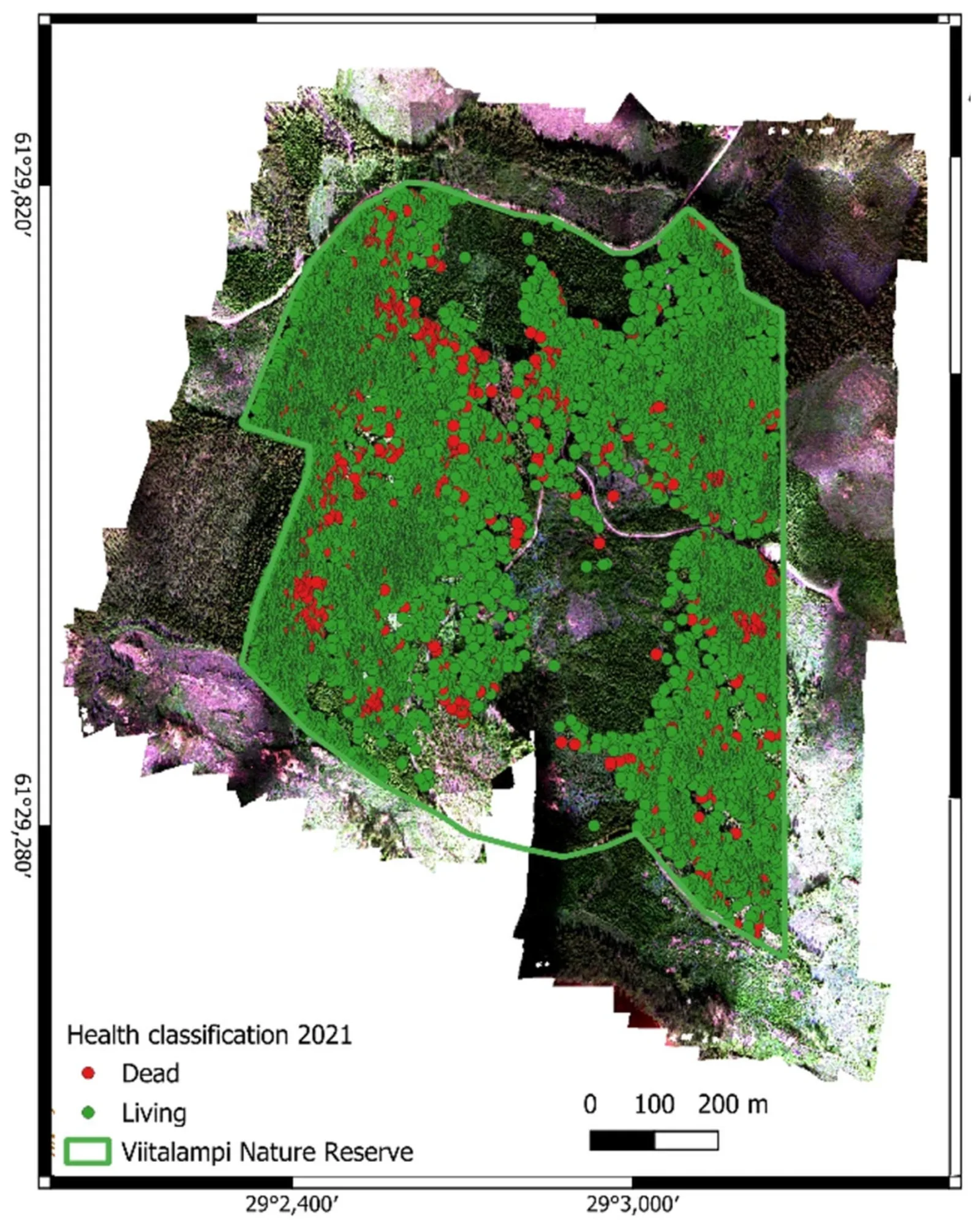
Spatial visualization of tree health assessment results.

Tree health assessment is commonly formulated as a multi-class classification problem, where each detected tree is assigned a health category based on spectral and structural information extracted from UAV imagery. This involves an individual tree-based workflow in which detected spruce trees are classified into healthy, declined, and dead classes using multispectral features and a Random Forest classifier. Unlike approaches relying solely on crown discoloration, their method also incorporated trunk-related symptoms, including resin flow, bark damage, and beetle entrance and exit holes, providing a more comprehensive assessment of tree vitality. The study further highlighted that distinguishing declined trees from healthy ones remains the most challenging stage of tree health assessment, whereas dead trees can be identified with considerably higher reliability. Figure 6 illustrates the output of tree health assessment as a georeferenced health map, where individual trees are assigned to health classes (living or dead) and displayed spatially across the forest stand. Such maps facilitate the identification of mortality hotspots and provide valuable information for monitoring forest condition and supporting management decisions [53,57].

#### 3.2.4. Counting

**Figure 7:**
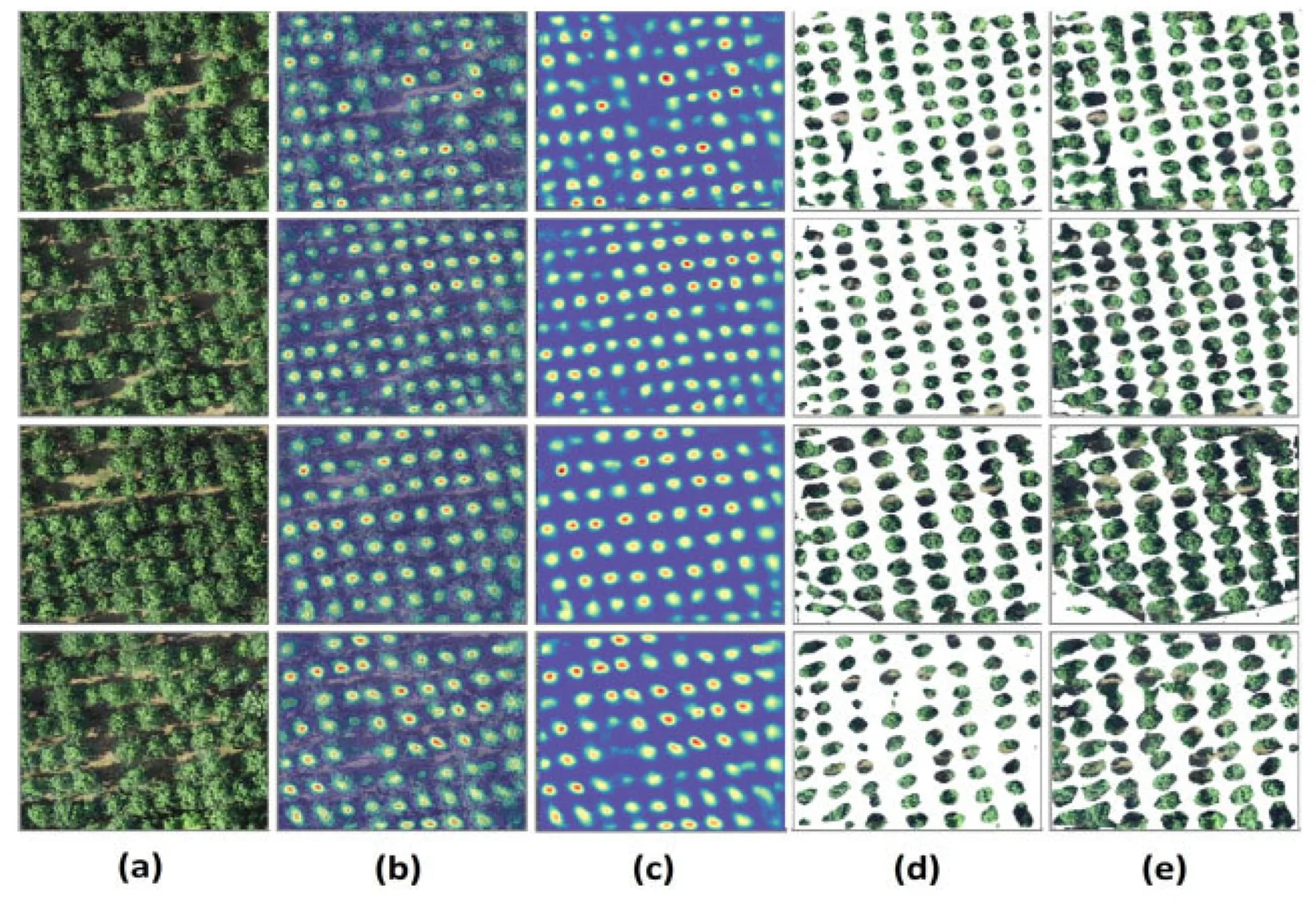
Illustration of tree counting strategies. (**a**) The input image. (**b**) The contour density map. (**c**) The filled contour density map. (**d**) The segmentation results with higher threshold. (**e**) The segmentation results with lower threshold.

Researchers tackle tree counting using two main strategies: count-by-detection and density-map regression. The first method simply tallies up individual predicted bounding boxes, a technique frequently applied to aerial imagery for mapping trees [40] or tackling similar visual hurdles like dense flower clusters [64]. Alternatively, density-map regression predicts a continuous surface density, integrating these values to estimate the final total. This approach really shines when managing large-scale tree inventories alongside crown segmentation [42], or within specialized networks built specifically for processing drone footage [65]. To gauge their accuracy, these counting studies typically rely on error metrics like MAE and RMSE, often pairing them with standard localization or segmentation scores when necessary. Figure 7 provides a conceptual comparison of the two dominant tree-counting approaches, highlighting the difference between explicit counting of detected tree instances and indirect estimation through density-map regression [65].

#### 3.2.5. Mapping/Georeferencing/Monitoring

**Figure 8:**
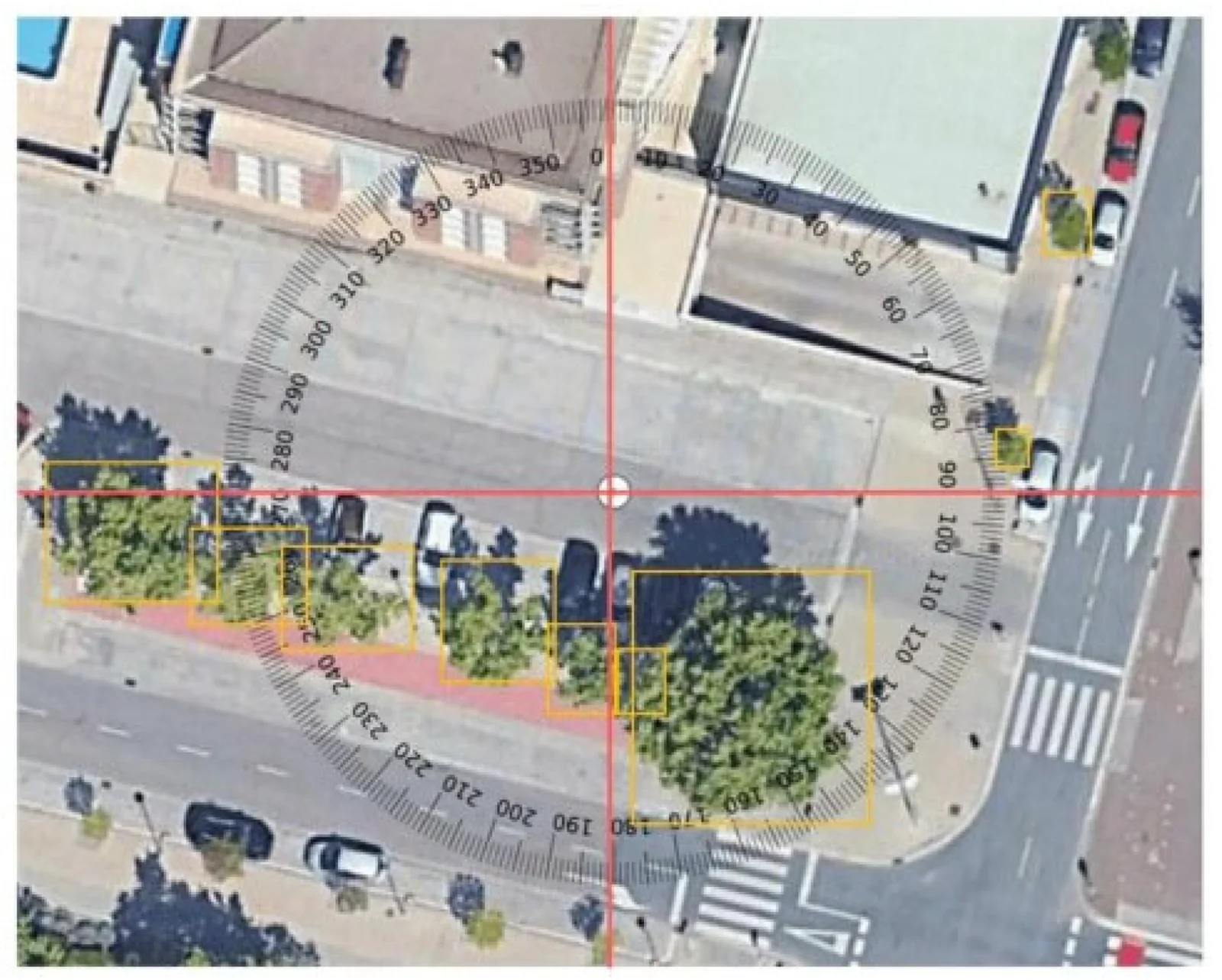
Example of a georeferenced spatial distribution map. Yellow boxes represent the predicted bounding box with the Deep Learning model.

Taking the analysis a step further, a substantial group of studies moves beyond simple per-image predictions to generate fully georeferenced outputs designed for real-world monitoring and inventory management. These practical applications range from plotting broad spatial distribution maps to tracking large-scale environmental changes and mapping isolated “trees outside forests.” Real-world examples of this shift include tracking long-term forest cover loss via satellite data [27] and developing dedicated workflows that tie urban tree detections directly to precise geographic coordinates [25], and UAV-based georeferenced mapping workflows based on visual image stitching [82]. Researchers are also leveraging powerful transformer models to map scattered trees across massive areas using high-resolution satellite imagery [35], while others focus on tracking the shifting dynamics of city street trees across multiple years through spatial linking [12]. Accurate geolocation is also a key component of autonomous UAV mapping systems, where object detection and localization are integrated with navigation and mission planning to support fully autonomous aerial operations [7]. Pushing this integration even further, certain frameworks successfully combine broad canopy mapping with precise individual tree counting into a single, cohesive pipeline. Figure 8 presents an example of a georeferenced spatial distribution map, demonstrating how computer vision outputs can be transformed into spatially explicit products for forest inventory, monitoring, and decision support [25].

#### 3.2.6. Notes on Multi-Task Pipelines and Scope

**Figure 9:**
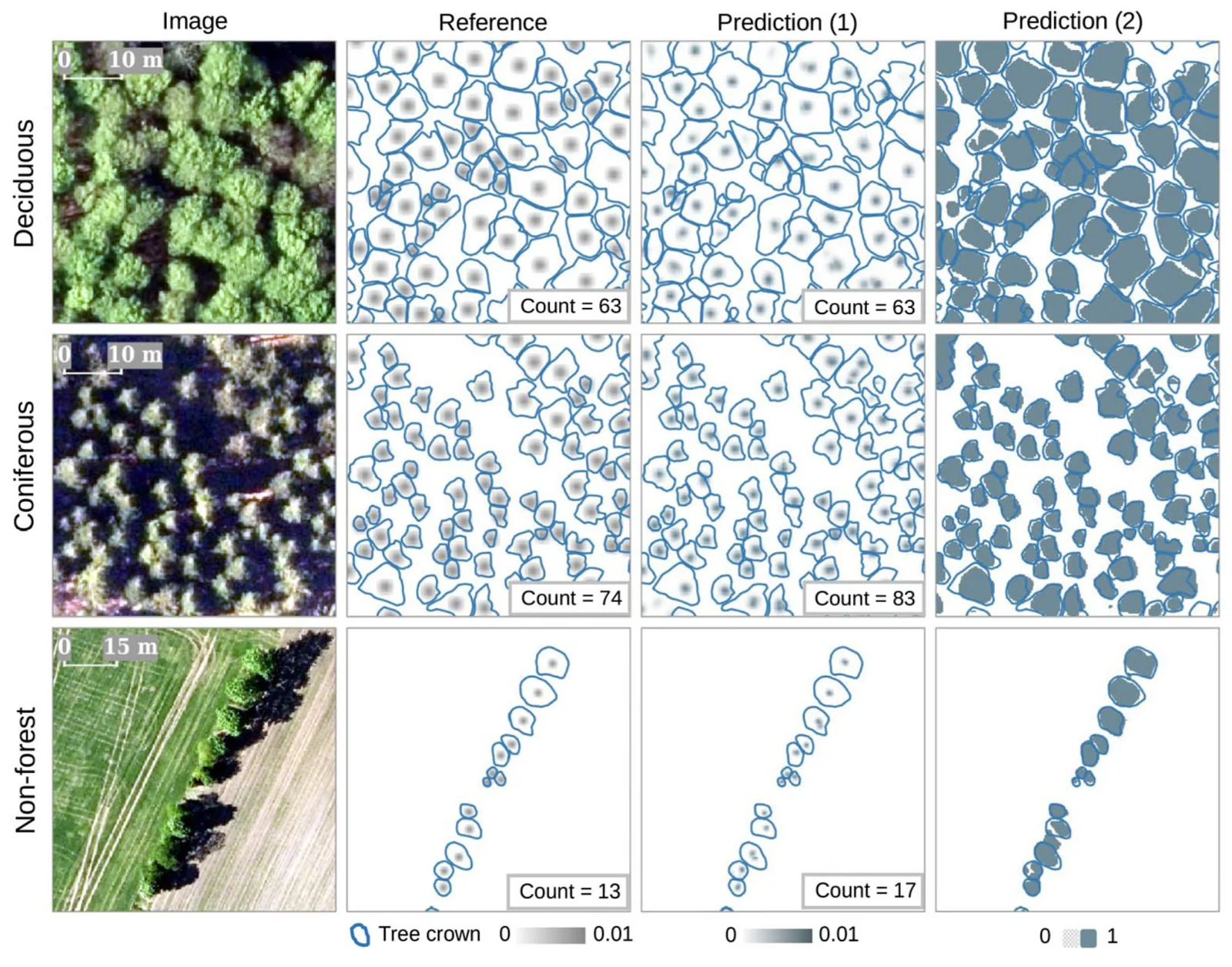
Examples from three different forest/landscape types: deciduous forest, coniferous forest, and nonforest areas. Reference shows the target labels, including the manual crown delineations (solid lines) and the Gaussian-blurred crown centroids. Prediction (1) shows the counting by density estimation results, and prediction (2) shows the crown segmentation results, both overlaid with the manual delineations.

To tackle complex environments, researchers frequently merge multiple objectives into a single workflow. A unified pipeline, for instance, might seamlessly integrate broad canopy segmentation with precise, crown-level counting [40,42]. Although our primary focus remains on urban trees, we also included a select few studies examining similar agricultural or vegetation targets. These related fields face identical hurdles, such as dense crowding, severe occlusion, and drastic scale variations and rely on the exact same toolchain of detection, segmentation, and counting algorithms [64,83,84]. Figure 9 presents an example of a unified multi-task workflow, illustrating how canopy segmentation, individual crown delineation, and tree counting can be integrated into a single computer vision pipeline [42].

### 3.3. Methodological Characteristics and Performance

Mapping urban trees today relies on a rich variety of deep learning algorithms, each fine-tuned to handle the unique spatial quirks of city environments. As detailed in Table 3, researchers primarily depend on advanced Convolutional Neural Networks (CNNs) and Vision Transformers (ViTs) to make sense of high-resolution images gathered from multiple sources. However, recent workflows for locating and detecting trees reveal a clear shift toward Transformer-based architectures, like DINO and Swin-T. These models excel at untangling overlapping branches while keeping track of the broader scene (detailed in Table 3).

An analysis of the performance metrics across these studies reveals that high-resolution datasets and the fusion of diverse data types are essential. By combining standard aerial or satellite photos with extra layers of information, such as Airborne Laser Scanning (ALS), street-level panoramas, or OpenStreetMap (OSM) data, systems consistently lock in strong detection rates and pin down tree locations with an impressive accuracy of around 60 cm [3,25,85]. All the underlying methodological parameters, dataset details, and core evaluation metrics for these various architectures are laid out in Table 3 below.

### 3.4. Data Sources and Spatial Resolution

Looking at the data driving these models, researchers rely on three main categories: drone footage, satellite imagery, or a multi-source fusion that blends top-down views with ground-level photos or specialized tools like LiDAR. Drones are a highly popular choice for capturing this information [9,24,89,90], with high-resolution satellites proving just as common across our evidence table [35,91,92,93]. Meanwhile, a smaller but innovative group of studies successfully mixes these various inputs to get the best of both worlds [5,25,31,94].

#### 3.4.1. UAV and VHR Aerial Imagery

Research heavily dependent on drones relies on RGB and occasional multispectral imagery to capture centimeter-level ground sampling distances (GSD). This extreme detail is vital for mapping precise crown boundaries and detecting tightly packed trees in cluttered urban environments [9,24,83,89]. Based on the reported data, the resolution of these aerial images’ spans from an incredibly sharp 3 cm per pixel [9] up to 30 cm per pixel [65]. Most other drone datasets fall comfortably between 4 and 20 cm [24,43,83,90], with some high-resolution aerial collections averaging around 15 cm per pixel [90]. Beyond standard drone flights, many researchers turn to airborne very-high-resolution (VHR) orthophotos. These offer a similar level of forensic detail, making them perfect for analyzing individual trees and establishing strict performance benchmarks [89,90,95]. Whenever a study zooms in on local scales to pinpoint single trees, trace out individual crowns, or identify specific species, these high-resolution data sources become essential [89,90,96].

#### 3.4.2. Satellite Imagery

Depending on the mapping goals and the sheer size of the area being studied, satellite-based research utilizes everything from commercial very-high-resolution (VHR) imagery to broader, coarser datasets [33,34,35,36]. Researchers frequently rely on established sensors like WorldView [34,36,91,92,93] and QuickBird [36,97,98], alongside medium-resolution constellations such as Sentinel [33,34,85,88] and Landsat [5,27]. The spatial resolution of these satellite images varies significantly: VHR setups often achieve an impressive 0.3 to 0.5 m [92], while large-scale tree mapping projects typically operate in the sub-meter to meter range, around 0.5 to 1.24 m [35]. At the broader end of the spectrum, some models effectively process multi-meter products at 2 m [91] or even three meters [98]. To squeeze even more detail out of the data, several workflows actively employ multispectral and pan-sharpened products, such as the sharpening 4 m multispectral imagery down to a crisp 1 m resolution, specifically to enhance urban canopy mapping [41].

#### 3.4.3. Mixed/Multi-Source Inputs

A smaller subset of research merges various data sources to boost spatial coverage and overall reliability. For instance, some studies integrate historical satellite photos with multispectral drone data [5], while others fuse aerial, satellite, and street-level imagery to accurately detect and map precise tree locations [25]. Researchers are also leveraging complex, multi-source streams, blending drone footage with satellite views and auxiliary sensors, to comprehensively assess urban greening [31]. To further enhance spatio-temporal modeling, certain satellite-focused projects even pull in complementary airborne data, such as pre-fire aerial LiDAR scans of tree crowns [94]. These hybrid pipelines expose a major practical hurdle in translating raw detections into final maps: dealing with drastically different resolutions and viewing angles. This inherent heterogeneity makes it incredibly difficult to compare performance metrics fairly, forcing researchers to meticulously align and normalize their diverse data streams [5,25,94].

#### 3.4.4. Seasonality and Acquisition Timing

Surprisingly, few studies in our dataset explicitly mention the specific seasons or dates when their imagery was captured. Among the exceptions, one urban canopy project specifically tracks the same area across summer, autumn, and winter [41], while another individual-tree study meticulously logs field and drone surveys conducted over several months during leaf-on conditions [89]. This seasonal timing is far from trivial; multiple researchers point out that shifting phenology, particularly the stark visual difference between leaf-on and leaf-off states, drastically impacts model performance, pushing the field to develop algorithms that generalize better across different times of the year [25,99,100].

### 3.5. Datasets and Annotation

**Figure 10:**
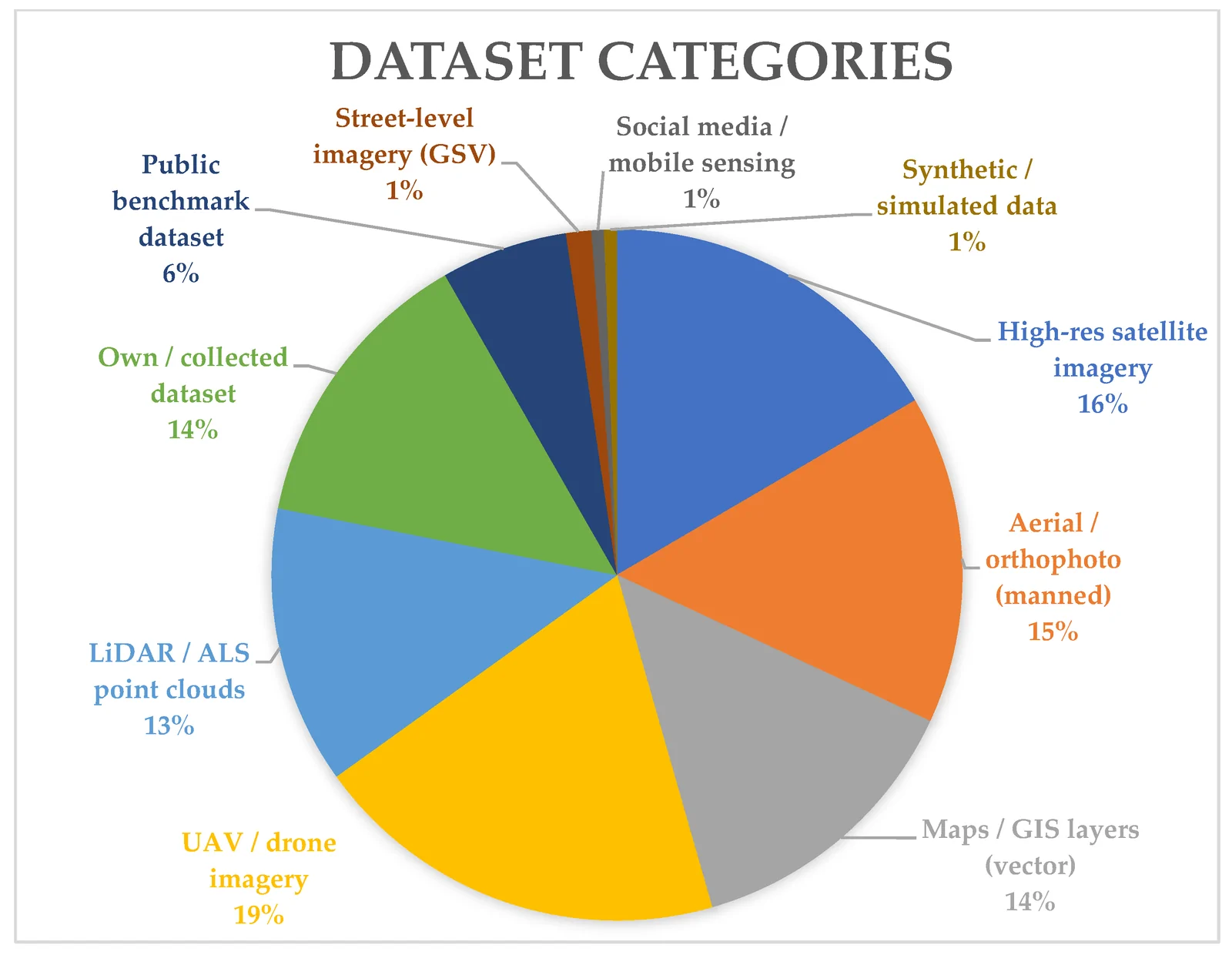
Distribution of dataset categories.

Researchers draw on a diverse array of datasets captured across various remote sensing platforms. These sources naturally fall into a few primary categories based on how the data is collected: drone footage, high-resolution satellite imagery, aerial orthophotos, and auxiliary inputs like LiDAR, a breakdown visually summarized in Figure 10 [5,24,25,89]. Drones are incredibly popular because their ultra-high spatial resolution makes it possible to accurately pinpoint single trees and trace intricate crown outlines even in the most cluttered urban settings [9,24,89]. Conversely, when the goal is to map vegetation across sprawling cityscapes, researchers typically turn to satellite imagery; while it lacks the extreme forensic detail of drone photos, it compensates by offering vastly broader spatial coverage [34,35,91].

A closer look at the dataset categories in Figure 10 reveals that UAV/drone imagery constitutes the largest data source (19%), followed by high-resolution satellite imagery (16%) and manned aerial/orthophoto imagery (15%). Maps/GIS layers and own/collected datasets each account for 14%, while LiDAR/ALS point clouds contribute 13%. Own/collected datasets account for 14% of the reviewed studies, indicating that a substantial number of researchers rely on locally acquired imagery and custom annotations for model development and evaluation. Public benchmark datasets represent a smaller proportion (6%), whereas alternative data sources including street-level imagery (1%), social media/mobile sensing (1%), and synthetic/simulated data (1%) remain rarely used in the current literature. To enhance structural information, many studies combine optical imagery with LiDAR-derived elevation products, such as Canopy Height Models (CHMs) or Digital Surface Models (DSMs), substantially improving crown delineation and three-dimensional scene interpretation [5,24].

Beyond the type of data, the sheer volume of imagery varies wildly across the field. Some projects rely on tight, custom-built collections featuring just a handful of annotated drone shots gathered over a single city district [9,89]. Conversely, broader initiatives scale up to process hundreds or even thousands of aerial and satellite captures. When publicly available benchmarks are used, datasets such as DeepForest and NAIP are among the most common resources for training and evaluation [39,100]. The way these datasets are labeled is entirely dictated by the specific task at hand. For standard object detection, researchers naturally default to drawing basic bounding boxes around individual trees or their crowns [12,25,37]. However, when the goal shifts to precise canopy mapping, segmentation models demand highly detailed pixel-level masks or intricate polygonal outlines [38,41,43]. For simpler inventory tasks like tree counting, basic point annotations marking the exact center of a tree often do the trick [42,84].

The incredible diversity in spatial resolution, dataset size, geographic spread, and annotation styles perfectly mirror the complex variety of urban forestry tasks, ranging from basic detection and counting to intricate crown segmentation and large-scale vegetation mapping.

### 3.6. Computer Vision Models and Architectures

**Figure 11:**
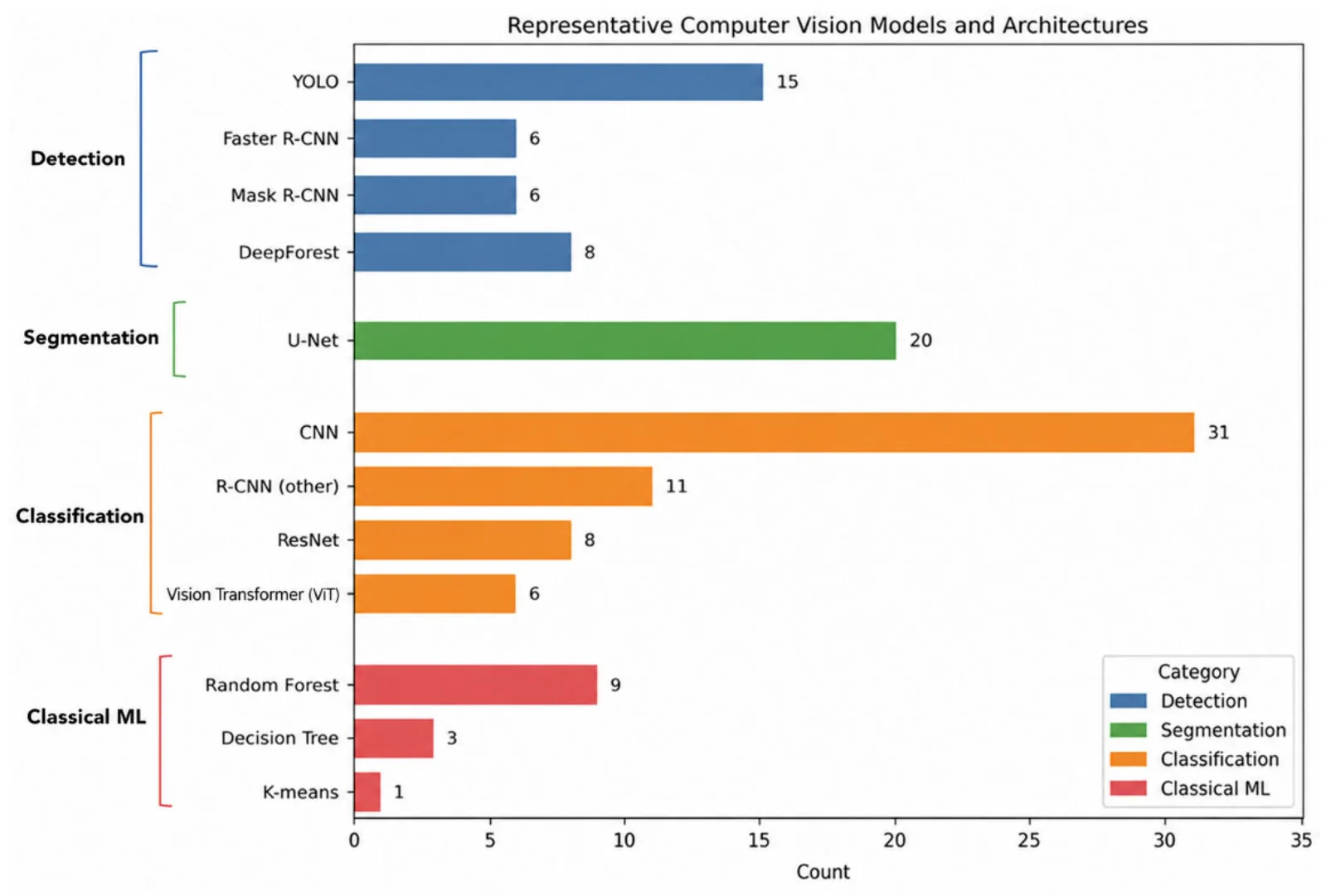
Representative computer vision models and architectures used in the reviewed studies.

The reviewed studies employ a broad spectrum of computer vision models that can be grouped into four major methodological categories: detection architectures, segmentation architectures, classification/feature extraction models, and classical machine-learning methods (Figure 11). CNN-based architecture, identified in 31 studies, represents the most frequently adopted family of models, reflecting the transition from traditional handcrafted approaches toward data-driven computer vision methods for urban tree analysis [38,41,43]. Among segmentation architectures, U-Net and its variants remain the most widely adopted models appearing in 20 studies. Their signature encoder–decoder structure, complete with skip connections, effectively preserves the crucial spatial details needed to delineate intricate tree crowns. Meanwhile, when the goal shifts to object detection, the YOLO family takes the lead. Appearing in 15 studies, these models deliver rapid inference speeds and manage massive image datasets with ease, making them ideal for spotting individual trees in densely packed urban environments [12,25,37,100].

Beyond single-stage detectors, two-stage frameworks from the R-CNN family remain highly relevant. Researchers frequently deploy Faster R-CNN and Mask R-CNN, each appearing in six studies, to tackle complex instance-level detection and crown delineation. These models are particularly effective when buildings, shadows, or overlapping branches introduce severe occlusions. Additionally, specialized frameworks like DeepForest have gained significant traction, appearing in eight studies thanks to their exceptional ability to generalize tree crown detection across drastically different aerial environments [42,64,101].

Within the classification category, CNN-based feature extractors remain dominant, while ResNet (8 studies) and Vision Transformer (ViT) architectures (6 studies) are increasingly adopted to learn more discriminative representations from high-resolution remote sensing imagery [35].

Despite the dominance of deep learning architectures, classical machine-learning methods remain relevant. Random Forest (9 studies), Decision Trees (3 studies), and K-means clustering (1 study) continue to be employed, particularly in hybrid workflows that combine handcrafted spectral or texture features with deep learning models [36,97,102]. Overall, Figure 11 summarizes the representative computer vision models according to their methodological role, highlighting the widespread adoption of CNN-based architectures together with the complementary use of detection frameworks, segmentation networks, and classical machine-learning methods.

### 3.7. Evaluation Protocols and Metrics

To ensure accurate and fair comparisons across varying urban environments, the reviewed studies rely on a diverse set of evaluation protocols and metrics, each carefully tailored to specific tasks like object detection, segmentation, and tree counting. Building a robust evaluation framework, complete with strict train, validation, and test data splits, is critical to this process.

When dealing with detection and localization tasks, where deep learning models pinpoint individual trees using bounding boxes, researchers naturally lean on standard object-detection metrics. The most common benchmarks used to measure success include Precision, Recall, the F1-score, and mean Average Precision (mAP) [12,35]. To push these models further, many studies report specific Average Precision thresholds, such as AP50, to rigorously evaluate their detection capabilities [24]. Overall detection accuracy also remains a primary yardstick, particularly when testing lightweight network architectures against the complex, cluttered backdrops of urban vegetation [23,25].

For workflows focused on canopy segmentation and outlining individual tree crowns, performance must be measured down at the pixel or instance level. To gauge the precision of these intricate models, researchers predominantly turn to Intersection over Union (IoU) and the Dice coefficient, often reporting them alongside Overall Accuracy and related per-class scores [21]. The F1-score, for instance, serves as a highly consistent tool for measuring exactly how well a predicted segmentation mask aligns with the actual ground truth [22].

When the objective shifts to counting trees or estimating density, whether predicting sheer numbers or generating continuous density surfaces, the evaluation naturally pivots toward regression and error-based metrics. In these scenarios, the Mean Absolute Error (MAE) and Root Mean Square Error (RMSE) are the gold standards for quantifying any discrepancies in the final count [42,65]. Finally, to prove that these automated mapping pipelines work in the real world, some studies go beyond basic pixel math to directly evaluate spatial alignment. By reporting the average positional error in meters, they can definitively validate just how accurately the detected trees are geolocated [25].

### 3.8. Comparative Performance Synthesis

To truly gauge how well modern deep learning models generalize and operate in the real world, we compared their reported performance across the two primary data sources: drones (UAVs) and high-resolution satellites. Naturally, making direct, head-to-head comparisons between these models is incredibly difficult, as the underlying studies rely on wildly different local datasets, shifting seasonal acquisition times, and inconsistent annotation protocols [5,12]. Despite these hurdles, synthesizing the core evaluation metrics, specifically mAP, F1-score, and IoU, still yields crucial insights into the broader performance trends driving each platform.

**Figure 12:**
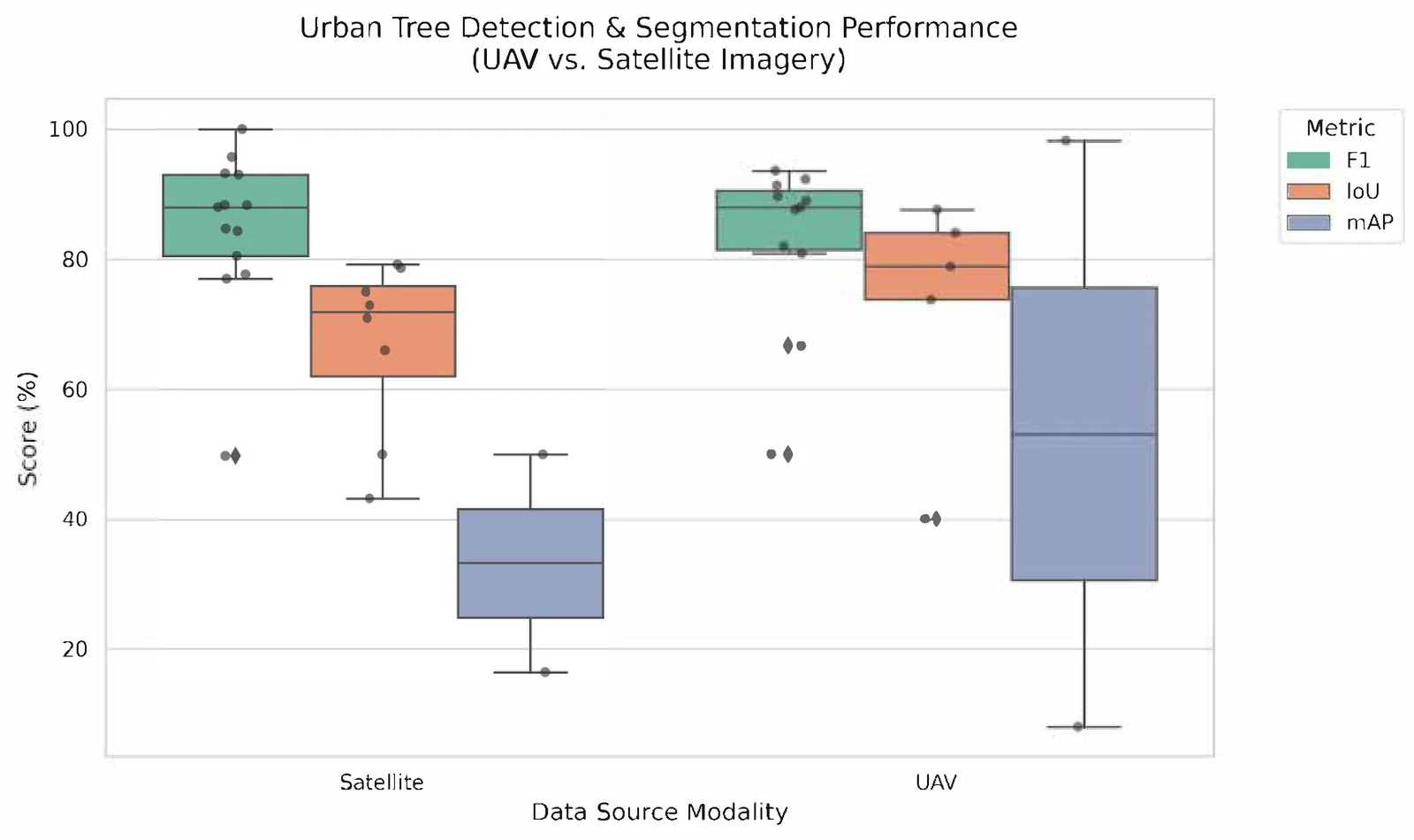
Comparative performance synthesis of urban tree detection and segmentation models.

As Figure 12 illustrates, models processing drone-derived imagery consistently outperformed their satellite counterparts, especially when the task demanded highly precise spatial boundaries [9,24,89]. For canopy and instance segmentation, UAV-based approaches achieved an average Intersection over Union (IoU) of roughly 73%, comfortably beating the 67% average seen in satellite models. This performance gap largely comes down to the extreme, centimeter-level detail (GSD) captured by drones, which cuts through the confusion of complex urban backgrounds and easily separates overlapping tree crowns [9,24].

This advantage naturally extends to object detection. When looking at mean Average Precision (mAP), drone imagery clearly takes the lead, allowing even some lightweight, single-stage models to push past 90% accuracy [12,24]. Satellite-based detection, on the other hand, frequently struggles with spatial misalignment and false negatives. These errors are typically triggered by harsh building shadows, skewed off-nadir viewing angles, and the generally lower spectral resolution found in cluttered streetscapes [5,8,98].

Interestingly, the F1-score, which balances Precision and Recall, remained highly competitive across both platforms, averaging between 82% and 84% [22]. However, satellite imagery showed a much wider variance in these scores. This fluctuation reveals a critical limitation: while high-resolution satellite data excels at broad vegetation mapping and simple presence-or-absence classification [34,35,91], it quickly loses its reliability when forced to delineate individual crowns or count specific trees in densely built urban areas [8].

Despite their lower average spatial accuracy for individual tree delineation, satellite imagery offers several important operational advantages. Very-high-resolution satellite data provide consistent acquisition conditions over large geographic areas without requiring flight permissions or site-specific deployment, making them well suited for regional and city-scale monitoring. Consequently, the choice between UAV and satellite imagery should depend on the application, with UAVs preferred for detailed local inventories and satellites better suited for large-scale and repeated monitoring.

### 3.9. Reproducibility and Openness

Reproducibility stands as the cornerstone of robust scientific research, yet it remains a formidable challenge in the field of automated urban tree mapping. Following established systematic reporting standards, the transparent sharing of datasets, analytical code, and research materials are vital for verifying results and driving the field forward. However, a synthesis of the current literature reveals deep-seated inconsistencies in open science practices. These gaps are largely fueled by the “black-box” nature of many deep learning architectures and a persistent shortage of high-quality, publicly available annotated datasets.

**Figure 13:**
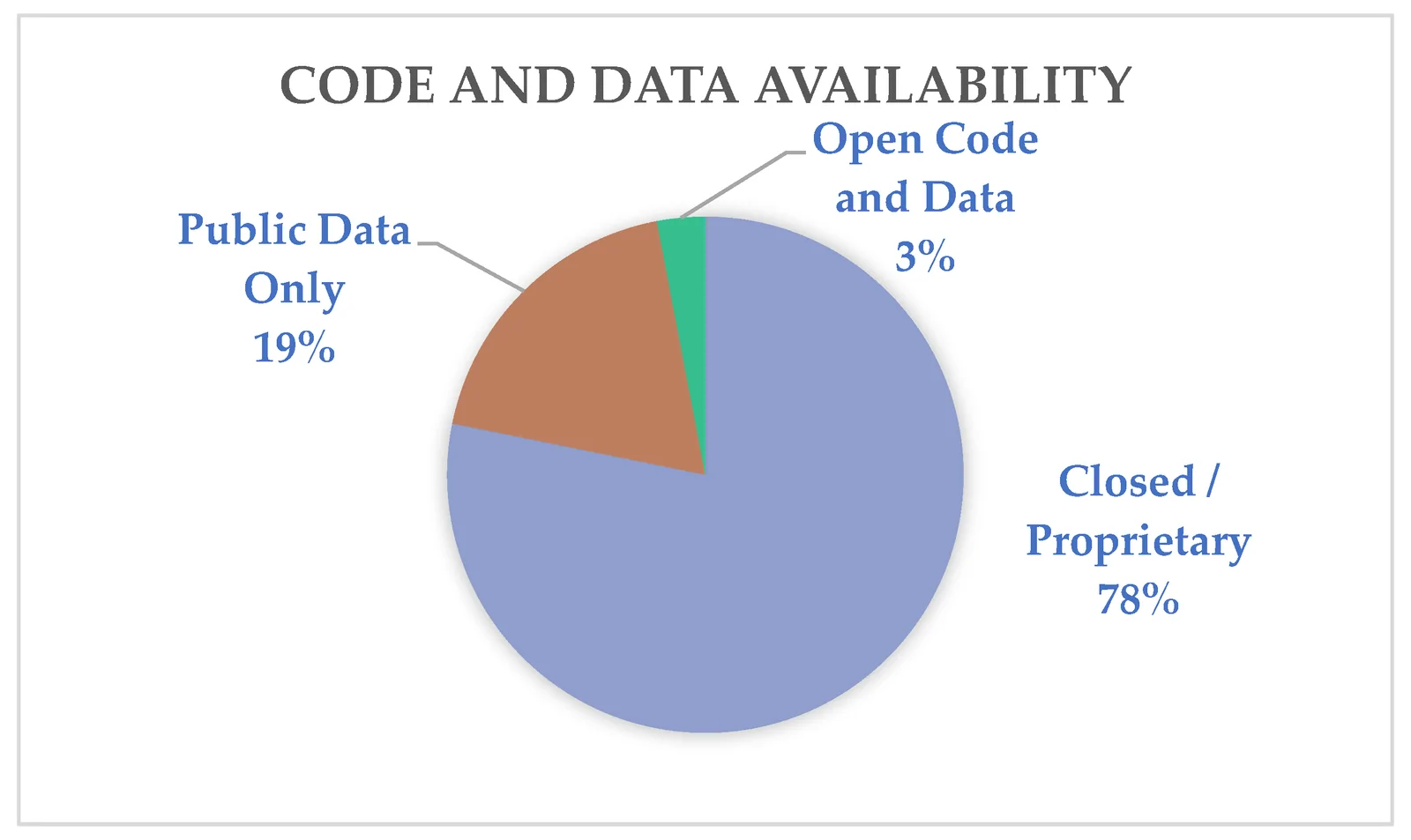
Distribution of the reviewed studies by reproducibility levels, categorized by the availability of open access datasets and source code.

The data summarized in Figure 13 reveals a striking gap in reproducibility across the current literature. Our analysis shows that the overwhelming majority of studies (78%) fall into the “Closed/Proprietary” category, relying on private data collections and keeping their source code hidden. These projects typically utilize custom UAV surveys or expensive commercial satellite imagery from sensors like WorldView and QuickBird [9,36,38]. Because these datasets are often bound by strict licensing agreements or privacy concerns, independent researchers find it nearly impossible to verify the reported performance metrics.

A smaller segment of the research (19%) is classified as “Public Data Only.” While these studies make use of accessible benchmarks like NAIP aerial imagery, DeepForest, or open access city inventories [12,25,101,103,104], they still fall short of full transparency by failing to release their custom model implementations or training code. Most concerning is the fact that only a tiny fraction of the field (3%) meets the “Open Code & Data” standard, providing both the raw data and the code needed for others to build upon their work [11,102].

Even when popular architectures like U-Net or YOLO are used, authors frequently omit the technical “fine print” that makes reproduction possible. Critical gaps, such as missing hyperparameter settings, undefined data augmentation steps, and vague descriptions of how data was split into training and test sets are common [34,42,91]. This lack of clarity does not just hinder reproduction; it slows down the development of models that can work across different cities. To move forward, the community must commit to releasing standardized urban benchmarks and sharing the analytical code behind every mapping pipeline.

### 3.10. Quality Appraisal Summary

In order to provide the scientific soundness of the research included, we performed a formal quality appraisal based on the principles of systematic review and were interested in the extent to which these studies reported findings in a transparent and strong manner. Particularly, issues that were investigated during this audit were the clarity of the dataset documentation, the validity of data-splitting strategies, the usage of competitive baselines, and ablation experiments.

Although technical specifications such as sensor types and ground sampling distance (GSDs) were generally well documented, an ongoing weakness was in terms of environmental context. In particular, most authors did not indicate the season of acquisition or phenological conditions of trees (leaf-on or leaf-off). It is a major oversight because seasonal changes have been found to radically change the detection accuracy and the generalization capabilities of a model in different climatic regions [42,86,87].

An urgent issue that can be identified during the appraisal is the approach to training and testing splits. Most studies give simple ratios of division, although they usually do not display transparency in terms of spatial segregations [34,91]. In high-resolution remote sensing, the mere randomization of overlapping image patches without the rigorous separation in geographical terms may result in data leakage, which artificially inflates the performance indices. Few serious studies have directly used spatial cross-validation or complete geographic isolation of the training and testing locations to avoid this bias [42,105].

On a positive note, standard baseline comparisons are prolific. New architectures are actively compared with stable architectures, such as U-Net, the YOLO family, or more classical object-based algorithms [38,91,106]. Moreover, the recent articles presenting complex alterations, e.g., attention mechanisms or multi-scale modules, always involve ablation experiments to support the introduction [8,43]. However, since such assessments are nearly always conducted on bespoke, non-public data, their external validity and reproducibility are constrained.

Moreover, there is a morphological selection bias that is observed in our appraisal. Most models are tested on the convenient locations of urban areas, e.g., trimmed parks or the well-laid avenues of streets, which gives the impression of near perfection. When used in uncontrolled areas that have trees of irregular shapes, overlapping, or deformed by diseases, then the accuracy of these models would reduce significantly. Such a propensity to experiment on perfected canopies blurs the actual working boundaries of the models in real life situations.

## 4. Discussion

### 4.1. Automated Individual Tree Detection and Precise Geolocation in Urban Environments

According to the recent literature, there is a definitive change in the approach to urban forestry, which is no longer a broad semantic segmentation of the green cover, but instead the accurate localization of individual trees. With intricate urban landscapes characterized by structural heterogeneity and thick, overlapping canopies, researchers are now placing greater emphasis on precise coordinate derivation rather than broad mapping. As an example, confidence-map regression has been used to a great success in the identification of tree centers in large regions [11]. Likewise, state of the art Vision Transformers, namely DINO and Swin-T models, have demonstrated exceptional performance at retrieving accurate bounding boxes, and are able to isolate individual crowns successfully even when they are physically interwoven [24,35].

To address the problem of the so-called urban canyons, when tall buildings produce deep shadows and cover trees with shadows, new workflows are exploring the use of multi-source data fusion. With aerial shots intersected by a ground-level panorama, such as the Google Street View, researchers can now triangulate and geolocate trees that are not visible at all, when looking down [25]. Moreover, Airborne Laser Scanning (ALS) combined with high-resolution orthophotos can be used to extract both accurate 3D structural information and crown widths [3].

To be very helpful in managing the city, these AI models should be scaled and able to operate in various regions. Although geographic area changes in domains continue to be a challenge, point-based detection systems are already implemented in various climate regions with great success [11]. The semi-supervised learning, as well as other more modern methods are also simplifying the process of adapting models trained in one continent to the completely different cityscapes [107].

Nonetheless, there is one serious obstacle, which is a strong geographical bias in training statistics. The existing studies are largely focused in China, Germany and the United States [12,32,33,100], making the models overfit the local lighting, tree species, and architecture. When moved to new contexts, these models often suffer a sharp drop in performance [11,12,35].

Another important limitation identified in this review concerns the biological representativeness of the datasets used to develop and validate deep learning models. Although most studies report high detection and segmentation accuracy, they are commonly evaluated on a relatively narrow range of urban forest communities and dominant tree species. Several investigations focus on species-specific datasets, such as *Ginkgo biloba* growing in urban environments, or on municipal tree inventories containing a limited number of genera, while others evaluate mixed forests dominated by only a few coniferous and broadleaf species, including *Pinus tabulaeformis*, *Populus nigra*, *Sophora japonica*, and *Ginkgo biloba*, or temperate European species such as *Picea abies*, *Pinus sylvestris*, *Abies alba*, *Fagus sylvatica*, and *Quercus* spp. [27,44,103,108]. Consequently, urban forests characterized by different species compositions, canopy architectures, successional stages, and disturbance regimes remain comparatively underrepresented in current benchmark datasets. This limitation is particularly relevant for monitoring mortality, pest outbreaks, and climate-induced decline in boreal coniferous forests, where species-specific physiological responses and crown morphology differ substantially from those observed in managed urban parks. Recent systematic reviews similarly emphasize that future UAV-based forest monitoring should incorporate broader ecological gradients, long-term observations, and biologically diverse benchmark datasets to improve model robustness and transferability across different forest ecosystems [46,49]. Addressing both the geographic and biological biases identified in the current literature will require globally distributed, publicly available benchmark datasets encompassing diverse climatic regions, urban forest communities, and dominant tree species. Such datasets, combined with Parameter-Efficient Fine-Tuning (PEFT) [95,101] and efficient spatial indexing schemes such as R-trees [35], could substantially improve the robustness and transferability of deep learning models.

Automated detection is worthwhile because it is connected to municipal infrastructure. Recent pipelines are now able to convert deep learning outputs (as points, boxes, or polygons) systematically to standard geospatial formats, such as Shapefiles or GeoJSON, to be used in QGIS (QGIS Development Team, https://qgis.org) and ArcGIS (Esri, Redlands, CA, USA, https://www.esri.com/arcgis, accessed on 24 July 2026) [7,24,25,35,82,109]. These systems are intended to refresh formal city tree registers directly or feed into interactive web maps and make huge datasets available to administrators and the general public [11].

Table 4 provides a comprehensive synthesis of the evaluation protocols used across modern tree detection systems, emphasizing a clear trend toward high-resolution data and metrics specifically designed for urban environments. By analyzing the methodological parameters from key studies [3,11,24,25,35], we can see a significant departure from basic pixel-wise classification in favor of precise object detection and geographic positioning. This evolution is mirrored in modern labeling techniques, which have moved beyond simple masks to include precise point-based coordinates for inventory databases and bounding boxes for individual crown isolation. Ultimately, the use of diverse metrics, ranging from standard F1-scores to specialized positional accuracy assessments, highlights how critical rigorous validation becomes when these AI-generated inventories are actually integrated into official municipal GIS platforms.

### 4.2. Automated Vegetation Segmentation in Fragmented Urban Canopies

The highlighted research proves that automated segmentation can successfully navigate the extreme fragmentation and spectral noise typical of city environments. Because urban canopies are often wedged between impervious surfaces, they frequently suffer from shadow occlusions and “mixed pixel” interference. To bypass these hurdles, researchers have introduced sophisticated architectures like CASNet, which uses global context attention to map fine-scale tree cover even from lower-resolution satellite data [33]. Similarly, the Double-Branch Multi-Scale Contextual Network (DB-MSC Net) leverages transformer blocks to keep local boundaries sharp against cluttered urban backdrops [8], while aux-HRNet focuses on preserving the integrity of highly fragmented green spaces within residential communities [87].

Beyond architectural improvements, these studies also tackle the prohibitive cost of city-scale data labeling. Since manual pixel-wise annotation is often too expensive, new frameworks are learning to thrive on sparse or incomplete crowd-sourced data. For instance, modified U-Net models now integrate OpenStreetMap (OSM) priorities with simple point labels to fill in the gaps [43]. Other workflows utilize Self-Organizing Maps (SO-UNet) to automate the creation of training samples through transfer learning [88]. By fusing multi-temporal imagery [41] or combining Sentinel-2 spectral bands with OSM geospatial layers [85], models can now account for seasonal changes and functional urban boundaries. This evolution marks a critical shift from testing on small, localized benchmarks [21,22] to deploying robust, multi-class assessments across entire metropolitan areas [86].

Table 5 reveals a methodological landscape built on a rich variety of labeling strategies and multi-sensor datasets, each specifically tuned for urban ecological tasks. This diversity is evident in how researchers choose their data: while some models excel at precise canopy delineation using high-resolution GF-2 or WorldView-3 imagery and pixel-level masks, others successfully deploy regression-style percentage cover or sparse point labels to map sprawling urban and peri-urban territories. The broad range of reported metrics from standard Overall Accuracy (OA) and IoU to RMSE for fractional cover highlights that monitoring fragmented urban greenery requires specialized evaluation criteria that can adapt to different spatial scales.

To ensure these semantic maps are actually useful for city planning, researchers rely on established geospatial ecosystems for storage and visualization. Pixel-wise predictions are typically exported as standard georeferenced rasters, such as GeoTIFFs, or converted into vector polygons. These files are then analyzed and managed within industry-standard GIS software, including ArcGIS (ArcMap version 10.2 and ArcGIS version 10.7; Esri, Redlands, CA, USA; Geospatial Platform|ArcGIS GIS Software for Business & Government), QGIS (QGIS Development Team, https://qgis.org), and ENVI (NV5 Geospatial Solutions, Hollywood, FL, USA; ENVI Remote Sensing Software for Image Processing & Analysis) [21,41,87]. For workflows that require object-based post-processing (OBIA), specialized tools like Trimble eCognition (Trimble Germany GmbH, Munich, Germany; https://geospatial.trimble.com/en/products/software/trimble-ecognition, accessed on 24 July 2026) are frequently used [86]. Furthermore, cloud-based platforms like Google Earth Engine (Google LLC, Mountain View, CA, USA; https://earthengine.google.com/) support large-scale visual assessments [88], while final datasets and source codes are increasingly shared through open repositories like GitHub (GitHub, Inc., San Francisco, CA, USA; https://github.com/), Zenodo (CERN, Geneva, Switzerland; https://zenodo.org/), or institutional drives to foster transparency [8,43].

### 4.3. Comparative Analysis: Object Detection vs. Semantic Segmentation

Choosing the right deep learning architecture is a pivotal decision in any urban forestry study. As established in the previous sections, object detection (localization) and semantic segmentation are designed to solve fundamentally different problems, each carrying its own set of technical trade-offs. To clarify these distinctions, the following section evaluates the relative strengths and weaknesses of both methodologies when applied to the structural complexities of urban landscapes.

As Table 6 highlights, choosing between object detection and semantic segmentation is a strategic trade-off rather than a simple technical preference. Object detection excels at isolating individual trees, providing the precise geographic coordinates needed to untangle overlapping crowns [24,35]. This makes it the ideal framework for updating municipal tree inventories, conducting health assessments, and populating GIS databases with vector points. However, because these models focus primarily on localization, they fail to capture the actual geometric footprint of the canopy.

Conversely, semantic segmentation is the preferred choice for measuring the total physical area and boundaries of green cover. This macro-level detail is essential for ecological research, such as mitigating the Urban Heat Island (UHI) effect or tracking neighborhood-level vegetation percentages [33,85]. The downside is that segmentation cannot reliably count individual trees within dense, continuous masses and is frequently prone to edge distortion from building shadows [8]. Furthermore, the pixel-wise annotation process is significantly more labor-intensive than the bounding-box or point-based labeling used for detection [11]. The architecture must align with the specific urban management goal: balancing the need for forensic individual localization against the demand for a comprehensive view of the total canopy area.

### 4.4. Roadmap for Future Research

Based on our systematic synthesis of the literature, Table 7 presents the following four-phase roadmap to bridge existing research gaps and achieve a robust, large-scale framework for automated urban tree mapping:

- Phase 1: Multimodal Fusion to Resolve Occlusions and Understory Mapping. Future architectures should move beyond 2D optical sensors to solve the “hidden understory” problem. Next-generation frameworks should directly fuse 2D instance semantics with 3D Airborne Laser Scanning (ALS) and LiDAR point clouds. This synergy is essential to pierce through building shadows, delineate sub-canopy structures, and provide the volumetric data required for accurate above-ground biomass estimation [3,8,101].
- Phase 2: Mitigating Domain Shift and Geographic Bias. To ensure global scalability, the research community must break out of its current “geographic echo chamber.” We recommend a collective effort to build and publish open access benchmark datasets that span diverse climatic zones and urban morphologies. Crucially, these future datasets must deliberately move away from “convenient sampling.” To build truly robust pipelines, the community must include morphological edge cases, such as diseased, structurally damaged, or naturally irregular canopies, ensuring that models learn to generalize about the messy reality of unmanaged urban forests. Enforcing standardized reporting of study locations will be critical for improving cross-city comparability and ensuring that models remain robust outside of traditional research hubs. Furthermore, the development of Parameter-Efficient Fine-Tuning (PEFT) is critical; it ensures that a network trained on one continent maintains accuracy when moved to a different climatic zone [24,95,103].
- Phase 3: Integration of Prompt-Guided Foundation Models. To resolve the long-standing trade-off between overlapping crown separation and exact area calculation, future pipelines should adopt hybrid, prompt-based architectures. By utilizing lightweight object detectors to generate bounding box “prompts” for heavy-duty Vision Transformers and Foundation Models (such as Segment Anything, TreePseCo, or DINO), researchers can achieve precise polygon delineation across vast areas without the need for exhaustive, site-specific fine-tuning [6,35,95].
- Phase 4: Transition to Multi-Task Ecological Assessment. The field must evolve beyond simple binary “tree-versus-background classification” detection. Future mapping pipelines should be designed for multi-dimensional analysis, including species classification, health monitoring, and the assessment of ecological stress. Developing these capabilities, even in the presence of sparse or noisy ground-truth annotations, is vital for turning raw spatial data into actionable insights for urban forest management [6,35].

### 4.5. Limitations of the Systematic Review

Although this systematic review adheres to established methodological guidelines, several inherent limitations in both the methodology and the synthesized literature must be addressed. First, the exclusion of non-English publications may introduce a linguistic bias, potentially overlooking relevant research published in other languages. Second, the extreme heterogeneity in sensor types, spatial resolutions, and evaluation protocols necessitated a narrative synthesis rather than a formal quantitative meta-analysis. The lack of standardized benchmarks and varying seasonal acquisition times [5,12] makes direct, head-to-head performance comparisons between models exceptionally difficult.

Furthermore, the current body of evidence exhibits a pronounced geographic bias, with research heavily concentrated in China, Germany, and the United States [2,6,12,33]. This concentration limits the global generalizability of the reported findings to different urban morphologies or climatic zones. Finally, widespread inconsistencies in open science practices persist; nearly 78% of the reviewed studies rely on closed or proprietary data [9,36,38], hindering independent verification and the broader reproducibility of these automated mapping models.

## 5. Conclusions

Based on the systematic analysis of 101 studies published between 2014 and 2026, the following conclusions can be drawn:

(1) Automated urban tree mapping has evolved from traditional detection methods toward accurate individual-tree localization and large-scale polygonal canopy mapping. UAV imagery generally provides higher spatial accuracy for individual tree detection and crown delineation, whereas satellite imagery remains indispensable for city- and regional-scale monitoring.

(2) Deep learning has become the dominant methodological paradigm. Although CNN-based architectures such as U-Net and YOLO remain the most widely adopted approaches, recent studies demonstrate an increasing shift toward Vision Transformers, hybrid architectures, and Foundation-Model-based workflows that improve both detection and segmentation performance.

(3) Despite these advances, important limitations remain. Most studies rely on proprietary datasets and closed-source implementations, limiting reproducibility, transferability, and large-scale validation across different geographic regions. Furthermore, the limitations of optical imagery highlight the importance of multimodal approaches integrating RGB imagery with LiDAR and other complementary data sources.

(4) Overall, the technological foundations for automated urban tree mapping are now well established. Future research should prioritize open science practices, standardized evaluation protocols, representative multi-regional benchmark datasets, and multimodal data fusion to improve robustness, reproducibility, and scalability across diverse urban environments.

## Figures and Tables

**Table 1:** Inclusion and exclusion criteria.

Inclusion Criteria	Exclusion Criteria
(1) Journal articles and review papers published in English;	(1) non-English publications;
(2) Studies addressing tree-related targets (e.g., individual trees, tree crowns, canopy, street/urban trees);	(2) Document types other than articles or reviews (e.g., editorials, short abstracts, posters, patents, theses, book chapters);
(3) Studies using UAV/drone imagery and/or high-resolution satellite imagery (as the main optical data source; additional modalities could be used when combined with optical imagery);	(3) Studies not focused on trees (or where trees were not a primary target);
(4) Studies applying artificial intelligence or machine-learning methods, with an emphasis on deep learning (e.g., CNN-based detectors/segmenters, transformer-based approaches);	(4) Studies relying solely on non-image data sources (e.g., field surveys only) or on remote-sensing modalities outside the scope of this review when optical imagery was not used (e.g., LiDAR-only studies without UAV/satellite optical imagery);
(5) Studies reporting sufficient methodological detail to interpret the workflow, including at least one of the following: dataset description, annotation/labeling scheme, evaluation protocol, or performance metrics.	(5) Studies without AI/ML-based analysis (e.g., purely manual interpretation or rule-based approaches without learning components);
(6) Studies involving the assessment of tree health or condition.	(6) Studies lacking sufficient methodological information or full-text availability to support extraction of key variables.

**Table 2:** Taxonomy of computer vision tasks in reviewed literature.

Task Category	Problem Formulation & Typical Outputs	Common Evaluation Metrics	Example References
Detection & Localization	Locating individual trees or crowns, typically producing bounding boxes or point coordinates using object detectors (e.g., YOLO, DETR).	Precision, Recall, F1-score, mAP	[6,12,25,35,36,37]
Segmentation & Crown Delineation	Pixel-wise mapping of tree canopies (semantic segmentation) or separating individual tree crowns (instance segmentation).	IoU, Dice coefficient, F1-score (per-class)	[6,38,39,40,41,42,43]
Tree Health Assessment	Assigning semantic labels to detected trees or tree crowns, including species identification, tree health status, damage severity, pest infestation stages, or vitality assessment using RGB, multispectral, hyperspectral, or structural information.	Accuracy, Precision, Recall, F1-score, Cohen’s κ	[44,45,46,47,48,49,50,51,52,53,54,55,56,57,58,59,60,61,62,63]
Counting & Density Estimation	Count-by-detection of predicted instances or integrating density-map regression surfaces for large-scale estimates.	MAE, RMSE	[40,42,64,65]
Mapping & Georeferencing	Translating predictions into spatial products, large-area mapping, and temporal change monitoring using geographic coordinates.	Task-dependent (often relies on underlying detection/segmentation metrics)	[12,25,27,35,40]

**Table 3:** Overview of deep learning architectures, methodological details, datasets, and performance metrics.

Reference	Task & Primary Algorithm	Methodological Details & Architecture	Used Dataset & Resolution	Performance Metrics
[3]	Tree Detection/Point-based	ALS data fused with orthophotos to update urban tree databases via structural feature extraction.	Helsinki pilot area (ALS + aerial orthophotos); 35,969 trees	F1 (location), Kappa (classification)
[8]	Street Tree Segmentation/Hybrid	DB-MSC Net combining parallel dilated convolutions and transformer blocks for multi-scale features.	GF-2 imagery street tree dataset; 10,000 patches	Overall Accuracy (OA), mIoU, PA, UA
[11]	Urban Tree Detection/Regression	Confidence-map regression to pinpoint tree center locations across multiple cities and climate zones.	NAIP tiles (California); 95,972 tree point annotations	AP, Precision, Recall, F-score, RMSE
[21]	Canopy Segmentation/ResUNet	Deep residual U-Net architecture for multi-class semantic segmentation focusing on tree boundaries.	ISPRS Vaihingen benchmark; 16 labeled tiles (RGB+IR)	IoU (~0.89), Dice coefficient (~0.94)
[22]	Canopy Segmentation/FCN	Fully Convolutional Network variants for pixel-wise mapping of continuous urban canopy cover.	Urban aerial orthophotos; 1394 images (0.48 m resolution)	Overall Accuracy (OA), segmentation accuracy
[24]	Individual Detection/Swin-T	Swin Transformer backbone combined with Faster R-CNN for bounding-box extraction and domain transfer.	Multiple test datasets (e.g., Campo Grande); 2736 crowns	Precision, Recall, F1 (IoU 0.5)
[25]	Detection & Geolocation/Triangulation	Multi-source fusion combining aerial/satellite with ground-level images to circumvent occlusions.	Custom urban dataset; >30 k ground trees, 10 k canopies	Positional accuracy (~60 cm), Detection rate (79%)
[33]	Cover Mapping/CASNet	Context-Aware Sub-pixel mapping network for fractional cover estimation using global attention.	Sentinel-2 + Google Earth labels; 0.13 billion samples	R2, RMSE, MAE
[35]	Crown Detection/DINO ViT	Detection Transformer with R-tree spatial indexing for bounding-box crown delineation over large areas.	TOF dataset; 330 patches (100 × 100 m), 23,643 annotated crowns	Precision, Recall, mAP
[41]	Canopy Segmentation/CNN	Double-Branch Convolutional Neural Network for binary semantic segmentation (tree vs. background).	Multi-temporal GF-2 blocks; 8768 train patches	Precision, Recall, F1-score, IoU
[43]	Tree Cover Segmentation/U-Net	U-Net adapted to learn from incomplete/sparse point labels guided by OpenStreetMap background priors.	Aerial orthophotos (Hamburg); 27 tiles (0.2 m), 11,366 trees	Balanced accuracy (~84%)
[85]	Green Space Mapping/Geospatial NN	Deep integration of high-resolution temporal spectral features with OSM geospatial vector data.	Sentinel-2 time series (Wuhan) + OSM polygons	Overall Accuracy (OA)
[86]	Multi-class Extraction/OBIA + U-Net	Object-based U-Net-DenseNet-coupled network refining pixel predictions into semantic land-use objects.	WorldView-3 imagery; 4761 subimage blocks (128 × 128 px)	Kappa (~0.72), Overall Accuracy (~82.7%)
[87]	Community Green Space/aux-HRNet	High-Resolution Network enhanced with auxiliary modules to preserve boundary integrity of fragmented patches.	GF-2 satellite imagery (Shanghai); 38,850 patches	IoU, mIoU, Precision, Recall, F1
[88]	Forest Segmentation/SO-UNet	Integration of Self-Organizing Maps with deep learning for automated training sample generation.	Sentinel-2 urban/peri-urban study areas; 5 land-cover classes	Overall Accuracy (OA), Kappa, IoU

**Table 4:** Summary of datasets and performance metrics for automated individual tree geolocation.

References	Task (Tree Detection)	Dataset	Images/Samples	Examples (Pictures)	Labels	Performance Metrics
[3]	Tree detection from ALS + aerial images (urban inventory)	Helsinki pilot area (ALS + orthophotos + urban tree DB)	8 km^2^; 35,969 trees	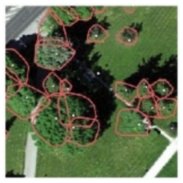 Tree crown delineation	Points/DB labels (tree locations; genera for classification)	F1 (location) + Kappa (classification)
[11]	Urban tree detection	NAIP tiles California cities	A total of 95,972 tree annotations	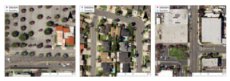 Tree detection results	Point annotations (tree locations) used to build confidence maps	AP, Precision, Recall, F score, RMSE
[24]	Individual tree detection	Multiple test datasets including Campo Grande	Campo Grande 17 images, 2736 crowns (other datasets also used)	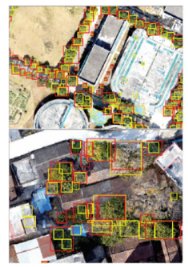 red: Faster R-CNN (Swin-T); yellow: Faster R-CNN (Resnet50).	Bounding boxes, IoU matching	Precision, Recall, F1 (IoU 0.5)
[25]	Urban tree detection + geolocation (multi-source)	Custom urban dataset combining ground-level + aerial/satellite	Ground-level: 4500 photos, >30,000 labeled trees; aerial/satellite: 979 images, 10,000 labeled canopies; resolution 10–60 cm	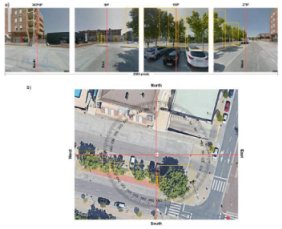 **(a)** Google Street View images from four cardinal directions. Yellow boxes indicate detected objects. **(b)** Google Maps satellite image. Yellow boxes indicate detected objects.	Mostly detection labels (tree locations/canopies; likely boxes/points depending on source)	Detection + positioning: 79% located with ~60 cm positional accuracy (reported)
[35]	Tree crown object detection (Transformer/DINO-based)	TOF dataset	330 image patches (100 m × 100 m), 23,643 annotated crowns; split: 300 train/15 val/15 test	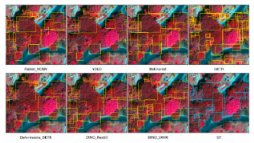 Yellow boxes indicate model predictions; cyan outlines represent ground-truth annotations.	Tree crowns annotated for detection (crown instances)	Precision/Recall-based evaluation (incl. IoU thresholding description)

**Table 5:** Summary of datasets, labeling strategies, and performance metrics in urban tree segmentation.

References	Task (Tree Segmentation)	Dataset	Images/Samples	Examples (Pictures)	Labels	Performance Metrics
[8]	Street tree segmentation	GF-2 imagery street tree dataset	10,000 patches 256 by 256 pixels. 8000 train, 1000 validation, 1000 test	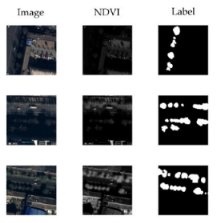	Pixel-level labels for street trees	OA, IoU, mIoU, PA, UA
[21]	Urban tree canopy mapping (semantic segmentation)	ISPRS Vaihingen (urban remote sensing benchmark)	16 labeled tiles used; each tile 5000 × 5000 px; 4-band RGB+IR	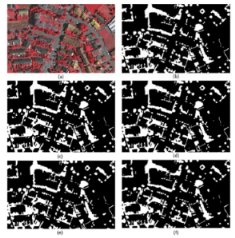 (**a**) original orthophoto, (**b**) 8-cm ground truth image, (**c**) 16-cm predicted tree canopy, (**d**) 16-cm ground truth image, (**e**) 100-cm predicted tree canopy, and (**f**) 100-cm ground truth image (white areas refer to tree canopy pixels; black areas refer to non-tree pixels).	Per-pixel land cover labels including tree class	Best results for tree class reported: IoU about 0.89 and Dice about 0.94
[22]	Urban tree canopy segmentation	Urban aerial orthophoto dataset	1394 images 3200 by 2400 pixels, 0.48 m resolution	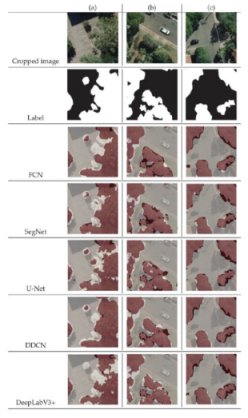	Manual canopy cover labels (pixel-wise)	OA and segmentation accuracy (as reported)
[33]	Urban tree cover mapping (segmentation or regression style)	Sentinel-2 plus Google Earth labels	0.13 billion samples. 1946 patches for validation	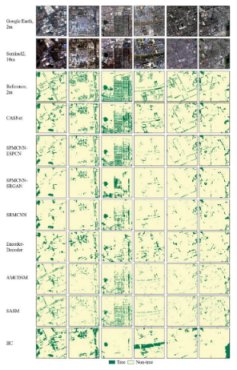	Tree cover values (percentage canopy cover)	R2, RMSE, MAE
[41]	Urban tree canopy mapping (binary semantic segmentation) from GF-2 imagery	Multi-temporal GF-2 dataset (blocks → patches)	60 image blocks (800 × 800) → patches (256 × 256): train/val/test 8768/2792/2792	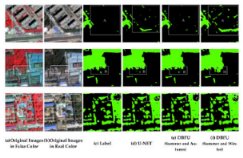	Binary mask labels (tree vs. background)	Precision, Recall, F1-score, IoU
[43]	Tree cover mapping from incomplete point labels (segmentation style output)	Hamburg, Germany orthophotos (District Hamburg-Nord)	27 tiles, each 5000 × 5000 px, 0.2 m per pixel, total 11,366 trees	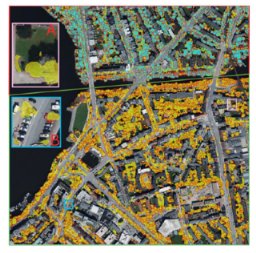 (A) Example of accurate detection of a blooming tree without misclassifying its shadow. (B) Example of successful detection of leafless trees.	Sparse point labels (incomplete) plus some fully annotated tiles	Balanced accuracy about 82% (sparse labels) and 84% (fully annotated)
[85]	Urban green space segmentation (not individual trees)	Wuhan city OSM + Sentinel-2 time series	Sentinel-2 sequence (2017–2020) + 1 test image (22 Aug 2020); OSM polygons (790 green belt, 100 wetlands, 10 water, 150 urban forest)	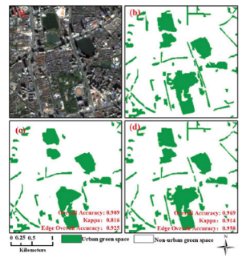 (a) Original Sentinel-2 image; (b) The ground truth reference; (c) The proposed neural network with SSP; (d) The proposed neural network with SPMP.	UGS classes from OSM polygons and POIs	Overall Accuracy (reported), (other metrics not clearly stated)
[86]	Urban forest resources extraction (multi-class segmentation/classification)	WorldView-3 imagery (urban land use classes including forest)	4761 subimage blocks, each 128 × 128 px	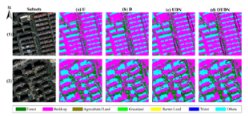 (**a**) the classification maps of the U algorithm in the subsets; (**b**) the classification maps of the D algorithm in the subsets; (**c**) the classification maps of the UDN algorithm in the subsets; and (**d**) the classification maps of the OUDN algorithm in the subsets.	Multi-class labels (urban land-use; includes forest/green classes)	Reported kappa about 0.721 and Overall Accuracy about 83% (for the shown experiment)
[87]	Urban community green space semantic segmentation (not individual trees)	GF-2 satellite imagery (Shanghai) + patch dataset	38,850 patches (512 × 1024); 7023 “community” images identified; 1000 labeled	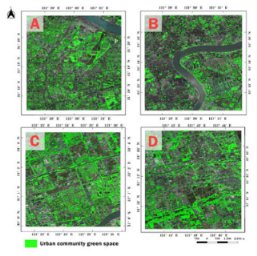 (**A**) is located in Baoshan District. (**B**) is located on the Bund, straddling Huangpu District and Pudong New Area. (**C**) is located in the new city of Pudong New Area. (**D**) is located in the suburban Minhang District.	Binary segmentation masks (labeled using LabelMe; split 60/10/30)	IoU, mIoU, Precision, Recall, F1
[88]	Forest/land-cover semantic segmentation (not individual trees)	Sentinel-2 urban + peri-urban study areas	8 images total (4 per study area, 2019)	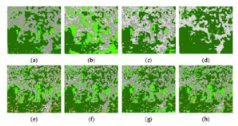 (**a**–**d**) UNet and (**e**–**h**) SO-UNet.	5 land-cover classes (includes forest class)	Overall Accuracy, Kappa, IoU

**Table 6:** Comparison of object detection and semantic segmentation methods for urban forestry applications.

Comparison Criterion	Recognition (Object Detection/Localization)	Segmentation (Semantic Segmentation/Masking)
1. Level of Detail and Data Type	Best aspect: Identifies each tree individually. The output consists of point coordinates or bounding boxes.	Best aspect: Calculates the exact area and physical boundaries of the canopy cover. The output is a pixel-wise mask or polygon.
2. Geolocation and Mapping Accuracy	Best aspect: Ideal for extracting the exact GPS coordinates of a specific tree to overlay on a map.	Worst aspect: Does not provide the exact coordinates of the trunk or crown center, showing only the general presence of a vegetation zone.
3. Overlapping Crowns Problem	Best aspect: Modern algorithms effectively separate densely packed street trees into distinct, individual objects.	Worst aspect: Merges adjacent trees into a single, continuous green mass, making individual tree counting impossible.
4. Dataset Annotation Effort	Best aspect: Annotating images with points or bounding boxes is fast and requires significantly fewer human-hours.	Worst aspect: Pixel-wise mask annotation is extremely labor-intensive, expensive, and difficult to collect at a city-wide scale.
5. Resilience to Urban Morphology (Shadows)	Best aspect: Detection based on crown centers suffers less from edge distortion caused by the shadows of tall buildings.	Worst aspect: Shadows create “mixed pixel” problems, resulting in jagged, noisy, and inaccurate segmented boundaries.
6. Scalability and Computation	Best aspect: Easier to scale to entire metropolises using spatial indexes (e.g., R-tree) to avoid double-counting across overlapping tiles.	Worst aspect: Ultra-high-resolution pixel-wise analysis over massive geographic areas demands immense VRAM and processing time.
7. Primary Practical Application	Best aspect: Individual tree inventory, updating municipal tree cadasters, and targeted monitoring of specific plantings.	Best aspect: Calculating the district-level greening percentage, analyzing the Urban Heat Island (UHI) effect, and macro-ecological planning.

**Table 7:** Roadmap for future research in automated urban tree mapping.

Phase	Identified Challenge	Proposed Strategy	Expected Outcome	References
Phase 1: Multimodal Fusion	Limitations of 2D optical sensors; building shadow occlusions and the “hidden understory” problem.	Direct fusion of 2D instance semantics with 3D Airborne Laser Scanning (ALS) and LiDAR point clouds.	Accurate delineation of sub-canopy structures and acquisition of volumetric data for above-ground biomass estimation.	[3,8,101]
Phase 2: Mitigating Domain Shift	“Geographic echo chamber,” domain shift, and “convenient sampling” that relies only on idealized, healthy canopies.	Building open access benchmarks that include morphological edge cases (diseased, irregular trees); enforcing standardized reporting; utilizing Parameter-Efficient Fine-Tuning (PEFT).	Global scalability, improved cross-city comparability, and model robustness in the messy reality of unmanaged urban forests.	[24,95,103]
Phase 3: Prompt-Guided Foundation Models	The trade-off between overlapping crown separation and exact area calculation; the high cost of site-specific fine-tuning.	Adopting hybrid architectures where lightweight object detectors generate bounding box “prompts” to guide heavy Foundation Models (e.g., SAM, TreePseCo, DINO).	Precise, large-scale spatial polygon delineation across vast areas without the need for exhaustive fine-tuning.	[6,35,95]
Phase 4: Multi-Task Ecological Assessment	The limitations of basic binary “tree-vs-background” detection and reliance on sparse/noisy annotations.	Transitioning mapping pipelines toward multi-dimensional analysis, including species classification, health monitoring, and ecological stress assessment.	Conversion of raw spatial data into comprehensive, actionable insights for urban forest management.	[6,35]

## Data Availability

No new data were created in this study. Data sharing is not applicable to this article.

## Abbreviations

The following abbreviations are used in this manuscript:

AI	Artificial Intelligence
ALS	Airborne Laser Scanning
AP	Average Precision
CHMs	Canopy Height Models
CNNs	Convolutional Neural Networks
DBH	Diameter at Breast Height
DSMs	Digital Surface Models
FCN	Fully Convolutional Network
GSDs	Ground Sampling Distances
IoU	Intersection over Union
LiDAR	Light Detection and Ranging
MAE	Mean Absolute Error
mAP	Mean Average Precision
MLS	Mobile Laser Scanning
NDVI	Normalized Difference Vegetation Index
OA	Overall Accuracy
OBIA	Object-Based Image Analysis
OSM	OpenStreetMap
PEFT	Parameter-Efficient Fine-Tuning
RMSE	Root Mean Square Error
SAM	Segment Anything Model
SfM	Structure-from-Motion
SVMs	Support Vector Machines
TLS	Terrestrial Laser Scanning
UAV	Unmanned Aerial Vehicle (drones)
UHI	Urban Heat Island
ULS	UAV Laser Scanning
VHR	Very-High-Resolution
